# scCODA is a Bayesian model for compositional single-cell data analysis

**DOI:** 10.1038/s41467-021-27150-6

**Published:** 2021-11-25

**Authors:** M. Büttner, J. Ostner, C. L. Müller, F. J. Theis, B. Schubert

**Affiliations:** 1 Institute of Computational Biology, Helmholtz Zentrum München, German Research Center for Environmental Health, Neuherberg, Germany; 2 Department of Statistics, Ludwig-Maximilians-Universität München, München, Germany; 3 Center for Computational Mathematics, Flatiron Institute, New York, NY USA; 4 Department of Mathematics, Technische Universität München, Garching bei München, Germany; 5 TUM School of Life Sciences Weihenstephan, Technical University of Munich, Freising, Germany

**Keywords:** Statistical methods, Transcriptomics

## Abstract

Compositional changes of cell types are main drivers of biological processes. Their detection through single-cell experiments is difficult due to the compositionality of the data and low sample sizes. We introduce scCODA (https://github.com/theislab/scCODA), a Bayesian model addressing these issues enabling the study of complex cell type effects in disease, and other stimuli. scCODA demonstrated excellent detection performance, while reliably controlling for false discoveries, and identified experimentally verified cell type changes that were missed in original analyses.

## Abstract (web summary)

Imbalance and loss of cell types is a hallmark in many diseases. Still, quantifying compositional changes in scRNAseq data remains challenging. Here the authors present scCODA, a Bayesian model to assess cell type compositions in scRNA-seq data.

## Introduction

Recent advances in single-cell RNA-sequencing (scRNA-seq) allow large-scale quantitative transcriptional profiling of individual cells across a wide range of tissues, thus enabling the monitoring of transcriptional changes between conditions or developmental stages and the data-driven identification of distinct cell types.

**Fig. 1:**
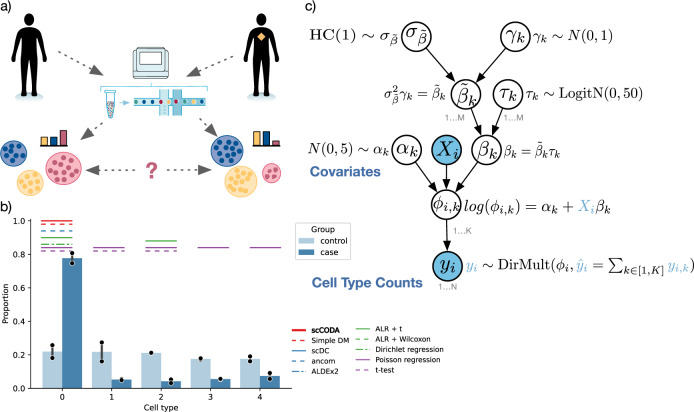
Compositional data analysis in single-cell RNA-sequencing data. **a** Single-cell analysis of control and disease states of a human tissue sample. Disease states reflect changes in the cell-type composition. **b** Exemplary realization of the tested scenarios with high compositional log-fold change and low replicate number (*n* = 2 samples per group). Colored horizontal lines indicate statistically detected compositional changes between case and control for different methods. The error bars denote the 95% confidence interval around the mean. **c** The scCODA model structure with hyperparameters. Blue variables are observed. *DirMult* indicates a Dirichlet-Multinomial, *N* a Normal, *logitN* a Logit-Normal, and *HC* a Half-Cauchy distribution.

Although being important drivers of biological processes such as in disease^1^, development^2^, aging^3^, and immunity^4^, shifts in cell-type compositions are non-trivial to detect using scRNA-seq. Statistical tests need to account for multiple sources of technical and methodological limitations, including the low number of experimental replications. The total number of cells per sample is restricted in most single-cell technologies, implying that cell-type counts are proportional in nature. This, in turn, leads to a negative bias in cell-type correlation estimation^5^ (Fig. 1a). For example, if only a specific cell type is depleted after perturbation, the relative frequency of others will rise. If taken at face value, this would lead to an inflation of differential cell types. Therefore, standard univariate statistical models that test compositional changes of each cell type independently may falsely deem certain population shifts as real effects, even though they were solely induced by the inherent negative correlations of the cell-type proportions (Fig. 1b). Yet, common statistical approaches currently applied in compositional cell-type analysis ignore this effect. For example, Haber et al.^6^ applied a univariate test based on Poisson regression, Hashimoto et al.^3^ a Wilcoxon rank-sum test, and Cao et al.^7^ proposed a method based on a generalized linear regression framework with a Poisson likelihood, all thus not addressing the issue of compositionality.

To account for the inherent bias present in cell-type compositions, we drew inspiration from methods for compositional analysis of microbiome data^8,9^ and propose a Bayesian approach for cell-type composition differential abundance analysis to further address the low replicate issue. The single-cell compositional data analysis (scCODA) framework models cell-type counts with a hierarchical Dirichlet-Multinomial distribution that accounts for the uncertainty in cell-type proportions and the negative correlative bias via joint modeling of all measured cell-type proportions instead of individual ones (Fig. 1c, Methods—“Model description”). The model uses a Logit-normal spike-and-slab prior^10^ with a log-link function to estimate effects of binary (or continuous) covariates on cell-type proportions in a parsimonious fashion. Since compositional analysis always requires a reference to be able to identify compositional changes^5^, scCODA can automatically select an appropriate cell type as the reference (Methods—“Automatic reference selection”) or uses a pre-specified reference cell type^11^. This implies that credible changes detected by scCODA have to be interpreted in relation to the selected reference. On top, the framework offers access to other well-established compositional test statistics and is fully integrated into the Scanpy^12^ ecosystem.

## Results

### scCODA performs best in a benchmark of synthetic datasets

We first performed comprehensive benchmarks on synthetic data across a wide range of scenarios (Methods—“Simulation”) that focused on scCODA’s primary application: the behavior of a single binary covariate that models the effect of a perturbation of interest in the respective scRNA-seq experiment. To detect statistically credible changes in cell-type compositions, we calculate the model inclusion probability for each covariate determined by the spike-and-slab prior (Methods—“Model description”). By using a direct posterior probability approach, scCODA automatically determines a cutoff on the posterior inclusion probability for credible effects that controls for the false discovery rate (FDR, Methods—“Spike-and-slab threshold determination”).

**Fig. 2:**
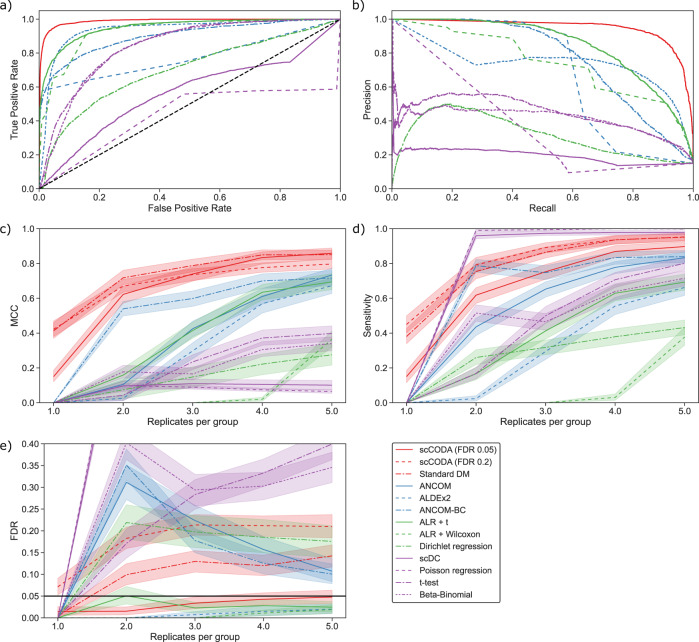
Comparison of scCODA’s benchmark performance to other differential abundance testing methods. Bayesian models (red), non-standard compositional models (blue), compositional tests/regression (green), non-compositional methods (purple). Shaded areas represent 95% confidence intervals. **a** Receiver-operating curve (*n* >1 samples per group). AUC scores are reported in (Supplementary Table 1)**. b** Precision-recall curve (*n* >1 samples per group). Average precision scores are reported in (Supplementary Table 1)**. c**–**e** Performance metrics with increasing number of replicates per group over all tested scenarios. In the case of *n* = 1 sample per group, only Bayesian methods are applicable, other methods cannot detect any changes. **c** Overall performance measured by Matthews’ correlation coefficient (MCC). **d** Sensitivity measured by true positive rate (TPR). **e** Precision measured by false discovery rate (FDR). The nominal FDR level of 0.05 for all methods (except scCODA with FDR 0.2) is indicated with a horizontal black line.

We compared scCODA’s performance to state-of-the-art differential compositional testing schemes from the microbiome field as well as all non-compositional tests recently applied to single-cell data (Fig. 2), all with a nominal FDR level of 0.05. In our synthetic benchmarks, we found scCODA to significantly outperform all non-Bayesian approaches in the regime of low-sample sizes across a wide variety of effects and experimental settings with an average Matthews’ correlation coefficient (MCC) of 0.64. Considering the number of replicates per group, the Bayesian models (scCODA and a standard Dirichlet-multinomial modeling approach; red lines in Fig. 2) had a considerable edge over all other methods in the common scenario with a low number of replicates per group, and increased their MCC further with the sample size (Fig. 2c). Other compositional non-Bayesian models such as ANCOM-BC^13^, ANCOM^14^, ALDEx2^15^, and additive log-ratio (ALR) transformed proportions combined with a *t* test (Methods—“Model comparison”) showed similar behavior, albeit with lower MCC. Non-compositional models, such as the Beta-Binomial model^16^, the scDC model^7^, or univariate *t* tests, (purple lines in Fig. 2) included more false positives with increasing effect size (Fig. 2d, e) and the number of replicates per group, highlighting the need for a compositional adjustment when modeling population data from scRNA-seq.

Looking at the false discovery rate (Fig. 2d), we could confirm recent findings that ANCOM and ANCOM-BC show increased numbers of false-positive results, especially in the low-sample setting^17,18^. Also, the standard Dirichlet-Multinomial model showed an average false discovery rate at almost twice the nominal level of 0.05. Only scCODA, ALDEx2, and the ALR-transformed statistical tests were able to accurately control for the false discovery rate in all scenarios. Of these methods, scCODA showed the best sensitivity (true positive rate; Fig. 2e) by a large margin. A more detailed look at the results in terms of effect size and the number of cell types is shown in (Supplementary Figs. 1–3).

When increasing the expected FDR level of scCODA to 0.2, the model sensitivity increased at the cost of a higher false discovery rate, which is controlled by the nominal FDR level (Fig. 2c–e).

Since non-Bayesian methods are not able to produce any results for the case of one sample per group due to a lack of degrees of freedom, we assumed no discoveries on these datasets, resulting in MCC, TPR, and FDR of 0. In contrast, Bayesian models adjust prior assumptions by the evidence from the data. Therefore, tests on one-sample data are possible, albeit with a strong influence from the choice of priors. Because scCODA gives equal prior probability to exclusion and inclusion of an effect (Methods—“Model description”), the selection of credible effects is driven by the data, even when the sample size is small. Supplementary Fig. 3 shows that Bayesian models can still detect some very strong effects (increase = 2000), even in the one-sample case.

We also performed sensitivity analysis by the receiver-operating characteristic and precision-recall curve (Fig. 2a, b and Supplementary Table 1). To allow for a fair comparison of frequentist and Bayesian methods, we only considered the case of more than one sample per group for all methods, since frequentist tests are not applicable in the one-sample case. Furthermore, we excluded the standard Dirichlet-Multinomial model from the comparison due to problematic thresholding. In both metrics, scCODA outperformed all other tested methods (AUC = 0.99; average precision Score=0.94). Most other compositional methods also showed adequate ability to accurately recover the true effects, while non-compositional methods were among the worst-performing methods.

While scCODA performs better than other methods in the low-sample case, we stress that analyses on datasets with larger sample sizes will always be less sensitive to outliers and variability in the data. To determine a reliable sample size for detecting effects of different strengths, we conducted a power analysis of our method.

### Power analysis to detect compositional changes

Since extensive replication of scRNA-seq experiments is still costly and hence rare, yet essential for studying compositional changes, we also investigated the sample size dependency of effect size and rarity of affected cell type on scCODA’s performance (Supplementary Fig. 4d–f). We performed a power analysis fitting a quasibinomial model (*R*^2^ = [0.937, 0.9377, 0.936] for FDR = [0.05, 0.1, 0.2], Methods—“Power analysis”) on true positive rate values to infer the required sample size to reach a power of 0.8 with a fixed FDR for varying log-fold changes (Supplementary Fig. 4d–f). We estimated that a relative change of 1 (log2 scale) in abundant cell types (e.g., 1000 out of 5000 cells) can be determined with five samples, while the same relative change requires between 20 and 30 samples in a rare cell type (e.g., 125 out of 5000 cells) at an FDR level of 0.2. Notably, large relative changes (log-fold changes of 4) in rare cell types could be detected with less than ten samples. While this implies that for many situations only a few replicates are necessary, we would advise to increase the number of samples when detection of compositional changes in rare cell types is relevant.

### scCODA identifies the FACS-verified decrease of B cells in supercentenarians

**Fig. 3:**
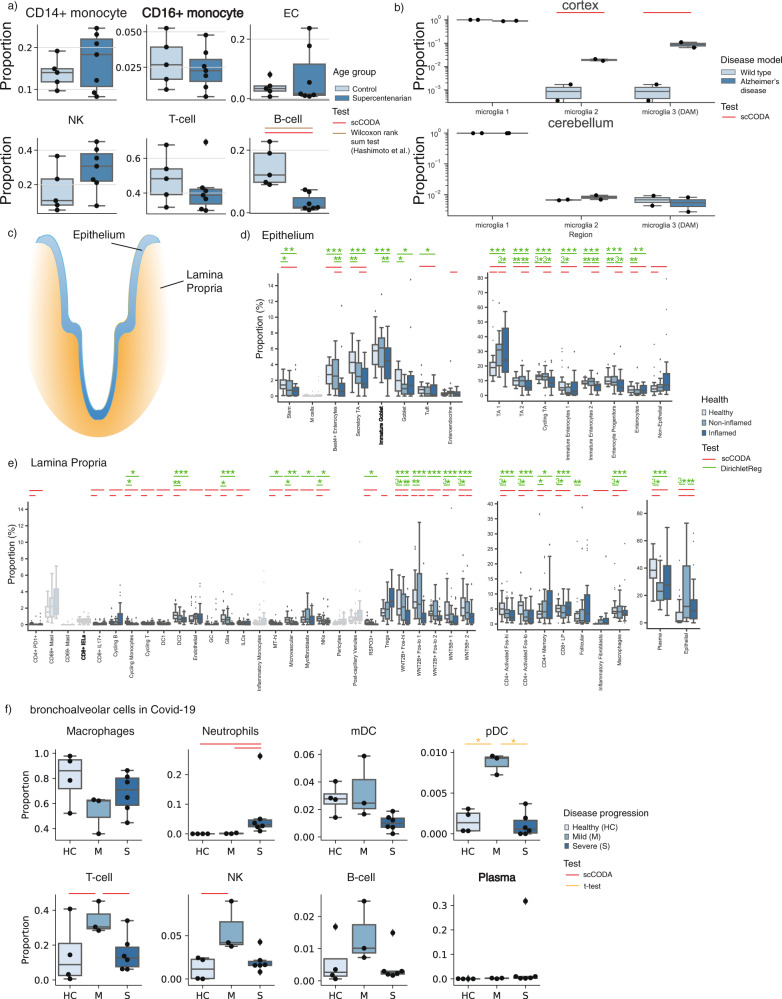
scCODA determines the compositional changes in a variety of examples. References are indicated in bold. **a** Boxplots of blood samples of supercentenarians (*n* = 7, dark blue) have significantly fewer B cells than younger individuals (control, *n* = 5, light blue), reference was set to CD16+ Monocytes, Hamiltonian Monte Carlo (HMC) chain length was set to 20,000 with a burn-in of 5000. Credible and significant results are depicted as colored bars (red: scCODA, brown: Wilcoxon rank-sum test (two-sided; Benjamini–Hochberg corrected)^3^). Results are in accordance with FACS data^3^. *P* values and effect sizes are shown in Supplementary Data 1**. b** Microglia associated with Alzheimer’s disease (AD) are significantly more abundant in the cortex, but not in the cerebellum^19^ (*n* = 2 in AD (dark blue) and wild-type (light blue) mice, respectively), HMC chain length was set to 20,000 with burn-in of 5000. *P* values and effect sizes are shown in Supplementary Data 2**. c**–**e** Changes in epithelium and lamina propria in the human colon^1^ in ulcerative colitis (UC) (*n* = 133 from 18 UC patients, 12 healthy donors). Credible and significant results are depicted as colored bars (red: scCODA, green: two-sided *t* test of Dirichlet regression coefficients). Stars indicate the significance level (*adjusted *P* < 0.05, **adjusted *P* < 0.01, ****adjusted *P* < 0.001; Benjamini–Hochberg corrected). **c** Epithelium and Lamina propria are distinct tissues, which are studied separately. **d** Compositional changes from healthy (light blue) to non-inflamed (medium blue) and inflamed (dark blue) biopsies of the intestinal epithelium, HMC chain length was set to 150,000 with burn-in of 10,000. *P* values and effect sizes are shown in Supplementary Data 3**. e** Boxplots of compositional changes from healthy (light blue) to non-inflamed (medium blue) and inflamed (dark blue) biopsies in the lamina propria, HMC chain length was set to 400,000 with burn-in of 10,000. *P* values and effect sizes are shown in Supplementary Data 3**. f** Boxplots of compositional changes in bronchoalveolar cells in COVID-19 patients (*n* = 4 healthy (light blue), *n* = 3 mild (medium blue), *n* = 6 severe (dark blue) disease progression)^4^. Credible and significant results are depicted as colored bars (red: scCODA, orange: *t* test (two-sided; Benjamini–Hochberg corrected)), references for scCODA: Plasma (all pairwise comparisons between conditions), FDR at 0.2. Stars indicate the significance level (*: adjusted *P* < 0.05, **adjusted *P* < 0.01, ***adjusted *P* < 0.001; Benjamini–Hochberg corrected), HMC chain length was set to 80,000 with a burn-in of 10,000. *P* values and effect sizes are shown in Supplementary Data 4**. a**, **b**, **d**–**f** In all boxplots, the central line denotes the median, boxes represent the interquartile range (IQR), and whiskers show the distribution except for outliers. Outliers are all points outside 1.5 times of the IQR.

Next, we applied scCODA to a number of scRNA-seq data examples^1,3,4,6,19^ (Fig. 3, Supplementary Figs. 5–9, and Supplementary Data 1). To confirm scCODA’s applicability on real data with known ground truth, we first considered a recent study of age-related changes in peripheral blood mononuclear cells (PBMCs)^3^, where cellular characteristics of supercentenarians (*n* = 7) were compared against the ones of younger controls (*n* = 5; Fig. 3a). The original study used a Wilcoxon rank-sum test and reported a significant decrease of B cells in supercentenarians, which is known from literature^20^. Moreover, the result was validated by FACS measurements. scCODA also identified B-cell populations as the sole affected cell type using CD16 + monocytes as a reference at an FDR level of 0.2. This suggests that scRNA-seq data indeed comprise enough information to study compositional changes, and that scCODA can correctly identify the experimentally validated age-related decrease of B cells even in low-sample regimes.

### scCODA detects staining confirmed increase of disease-associated microglia in Alzheimer’s disease on few replicates

Second, we analyzed the compositional changes of three microglia cell types in an Alzheimer’s disease (AD) mouse model^19^ (Fig. 3b and Supplementary Data 2). Here, the number of replicates of sorted cells from cortex and cerebellum was low (*n* = 2 per group), thus challenging standard statistical testing scenarios. In the cortex, scCODA identified statistically credible changes both in microglia 2 and disease-associated microglia (DAM) using the most abundant tissue-resident microglia 1 as reference cell type, or a credible change in microglia 1 when using one of the other two types of microglia as the reference. By contrast, scCODA detected no statistically credible change in the cerebellum, which is known to be unperturbed in AD. Keren-Shaul et al.^19^ quantified the increase of DAM in the cortex of the AD mouse model via staining. While DAM localize in close proximity to amyloid-beta plaques and show a distinct inflammatory gene expression pattern, microglia 2 tend to represent an intermediate state between DAM and homeostatic microglia 1^19^ (Supplementary Fig. 6). Therefore, our analysis with scCODA supports the contribution of DAM in AD. For comparison, ANCOM identified all three types of microglia as significantly changing in the cortex, and none in the cerebellum.

### scCODA scales to large sample sizes and cell-type numbers

We next analyzed compositional changes of cell types in single-cell data from patients with ulcerative colitis (UC) compared to healthy donors^1^. Here, biopsy samples from the epithelium and the underlying lamina propria (Fig. 3c, Supplementary Data 3, and Supplementary Fig. 7) were enzymatically separated and subsequently analyzed with scRNA-seq, resulting in 51 cell types from 133 samples. The epithelium and the lamina propria represent two different compartments and were tested separately. However, some epithelial cells ended up in the lamina propria samples and vice versa. For testing, we summarized these cells as nonepithelial in the epithelium and as epithelial in the lamina propria (Fig. 3d, e). We then reanalyzed the data with the Dirichlet regression model used in Smillie et al.^1^, leading to more statistically significant results compared to the original publication. Similar to the Dirichlet regression model, scCODA identified several statistically credible cell-type changes in healthy tissue compared to both non-inflamed and inflamed tissue in both epithelium and lamina propria at an FDR level of 0.2, using Immature Goblet cells and CD8 + intraepithelial lymphocytes (IELs) as automatically selected reference. Notably, we tested scCODA with different reference cell types (Supplementary Fig. 8), and did not detect credible changes for CD8 + IELs in the lamina propria with any reference cell type, backing up that CD8 + IELs are a good reference that does not change with respect to any other cell type. In the epithelium, both Dirichlet regression and scCODA identified significant and statistically credible changes, respectively, in the absorptive and secretory lineage, but scCODA also identified an increase in enteroendocrine cells. For comparison, ANCOM only identified significant changes in M cells (healthy vs inflamed) and enteroendocrine cells (healthy vs non-inflamed). M cells are lowly abundant and only 3 out of 16 inflamed samples had more than ten cells. When we compared the M-cell-positive subset of inflamed samples to healthy samples, though, M cells were indeed credibly increased. In the lamina propria, B-cell subpopulations showed several changes, e.g., a decrease of plasma B cells with disease (validated with stainings in Smillie et al.^1^), and an increase of follicular B cells. Moreover, consistent with our simulation studies demonstrating scCODA’s higher sensitivity for lowly abundant cell types, scCODA uniquely detected statistically credible changes in several low-abundant immune cell populations. For instance, scCODA identified regulatory T cells (T_reg_) to be more abundant in UC patients which is consistent with other studies^21^. Smillie et al. combined the results of their Dirichlet regression with two non-compositional tests, Fisher exact test and Wilcoxon rank-sum test, to identify absolute changes in each population independently. Using such a two-stage procedure, Smillie et al. also reported changes in the low-abundant cell types such as T_reg_ cells. For comparison, ANCOM only identified significant changes in inflammatory fibroblasts (healthy vs inflamed), epithelial cells and pericytes (healthy vs non-inflamed), while all cell types in non-inflamed vs inflamed were reported as significantly changing. In contrast to Dirichlet Regression, scCODA reported credible changes in inflammatory fibroblasts (IAFs) for the healthy vs. inflamed case. Similar to M cells in the epithelium, IAFs form a responding subgroup within the inflamed donor group: While the cell type was almost absent in the control group, 13 out of 24 UC patient samples had more than five cells, indicating that ANCOM and scCODA are more likely to detect lowly abundant or absent cell types, where changes manifest in a subset of samples. On the other hand, only 10 out of 24 samples in the non-inflamed group had more than five IAFs, which was not enough for both methods to detect a credible change. We tested scCODA’s performance to detect compositional changes when only a subset of samples exhibits a response (Supplementary Fig. 9). Consistent with the observations on IAFs, lowly abundant cell types show credible changes only when at least half of the samples change upon stimulation.

### scCODA detects cell-type changes in COVID-19 patients that were not detected with non-compositional tests but confirmed in larger-scale studies

Next, we reanalyzed a recent COVID-19 single-cell study comparing compositional changes of major cell types in bronchoalveolar lavage fluid between healthy controls (*n* = 4), severe (*n* = 6) and moderate (*n* = 3) COVID-19 cases^4^ using plasma as manually selected reference (Fig. 3f and Supplementary Data 4). The study originally reported significant differential changes in pDC’s in healthy vs moderate and moderate vs severe, respectively, depletion in mDCs in severe vs healthy, and depletion of T cells in severe cases vs. moderate cases using a *t* test without multiple testing correction. Correcting for multiple testing resulted in only pDC’s reported as significantly changing in healthy vs mild and mild vs severe cases, respectively. scCODA confirmed the differential change in T cells, and identified a credible increase in NK cells between mild vs healthy cases, credible depletions of T cells between moderate vs severe cases, as well as a credible increase of neutrophils in healthy and moderate vs severe at an FDR level of 0.2 using Plasma as reference. For comparison, ANCOM identified significant changes in mDCs between healthy and moderate, as well as neutrophils between healthy and moderate vs severe at alpha=0.2, respectively. The correlation of T-cell abundances with severity is well established and has been used as risk factors for severe cases^22,23^. A decrease of NK cells with COVID-19 severity was observed between recovered and diseased patients^23^ in PBMC through FACS analysis. Finally, higher neutrophil proportions have been associated with severe outcomes^24^ and are suspected to be the main drivers of the exacerbated host response^25^, further confirming scCODA’s findings.

### scCODA accounts for the negative correlation structure for compositional changes and shows fewer false positives

Our final analysis considered a longitudinal scRNA-seq dataset from the small intestinal epithelium in mice, studying the effects of *Salmonella* and *Heligmosomoides polygyrus* infection on cell-type composition^6^. In contrast to the original Poisson regression data analysis^6^, scCODA found only a single statistically credible increase in Enterocytes in *Salmonella* infected mice for an FDR level of 0.2 (Supplementary Fig. 10 and Supplementary Data 5). In addition, the Poisson model identified Tuft cells to be significantly affected after three and ten days of infection with *H. polygyrus*, while Enterocytes, Goblet, and early transit-amplifying cells were found to change significantly only after ten days of infection (Supplementary Fig. 10). All these changes could not be confirmed by scCODA at an FDR level of 0.2. For comparison, ANCOM did not find any significant changes for all three conditions, confirming its lack of power for datasets with few samples.

## Discussion

In summary, using a comprehensive set of synthetic and scRNA-derived compositional datasets and application scenarios, we established scCODA’s excellent performance for identifying statistically credible changes in cell-type compositions, while controlling for the false discovery rate. scCODA compared favorably to commonly used models for single-cell and microbiome compositional analysis, particularly when only a low number of experimental replicates are available. We believe this is due to the Bayesian nature of the model as it adequately accounts for the uncertainty of observed cell counts, automatically performs model selection, and does not rely on asymptotic assumptions. scCODA not only correctly reproduced previously discovered and partially FACS-verified compositional changes in recent scRNA-seq studies, but also identified additional cell-type shifts that were confirmed by independent studies, including T_reg_ cell enrichment in UC patients and neutrophils increase in severe COVID-19 cases. Using synthetic benchmarks, we confirmed that standard univariate tests, such as Poisson regression models, Beta-Binomial regression, or *t* tests are inadequate for cell-type analysis, since they do not account for the compositional nature of the data. While log-ratio transforms from compositional data analysis (such as the ALR used here) can partially mitigate these shortcomings, our Bayesian scCODA framework provided substantial performance improvements across all tested scenarios and is particularly preferable when only few replicates are available. Other methods from the field of microbiome data analysis, such as ANCOM and ANCOM-BC, showed similar detection power, but could not adequately control the false discovery rate in the low-sample regimes.

While scCODA shows excellent performance in our simulation studies and applications, the current modeling framework possesses several limitations. In its present form, the scCODA framework requires pre-specified cell-type definitions which, in turn, hinge on statistically sound and biologically meaningful clustering assignments. In situations where crisp clustering boundaries are elusive, for instance, due to the presence of the transient developmental processes underlying the data, joint modeling of different resolution hierarchies^26^ or modeling compositional processes^27,28^ may help account for such continuities changes. Furthermore, scCODA assumes a log-linear relationship between covariates and cell abundance, which may be mis-specified in some cases. Thus, scCODA may benefit from incorporating appropriate transformation models for the covariate data to achieve approximately log-linear relations. In its current form, scCODA does not model or infer any dependency structure among the cell compositions beyond the ones induced by the compositional effects. While more complex dependencies could, in principle, be included via additional hyperpriors, this would considerably increase the computational complexity and would require more efficient inference algorithms. Finally, scCODA does not model the response variability within a condition and thus cannot detect heterogeneities between samples in response to treatment or donor variability, as, e.g., in the data of UC patients^1^. This could be addressed by adding a novel covariate to inspect subsets of the data.

Overall, we believe that our scCODA framework offers an ideal starting point to model such advanced processes thanks to its hierarchical and extendable nature.

## Methods

### Model description

We seek to identify the credibly associated covariates X^NxM^ to observed cell counts Y^NxK^ of *K* cell types measured in a single-cell experiment with *N* samples and *M* covariates. We address this question with a Bayesian generalized linear multivariate regression framework using a Dirichlet-Multinomial model with a log-link function to account for the compositional nature and uncertainties in the observed data. Effects between covariates *m* and cell types *k* are hierarchically modeled using individual, normally distributed effects ${\gamma }_{m,k}$ with a covariate-specific scaling factor ${{{{{{\rm{\sigma }}}}}}}_{m}^{2}$^29,30^. For automatic model selection and identification of credibly associated covariates and affected cell types, we utilize a logit-normal prior as a continuous relaxation of the spike-and-slab prior^10^ resulting in the following hierarchical model:

1 $$Y \sim {{{{{\rm{DirMult}}}}}}(\phi ,\bar{y})$$

2 $$\log (\phi )={{{{{\boldsymbol{\alpha }}}}}}+X\beta$$

3 $${\alpha }_{k} \sim {{{{{\rm{N}}}}}}(0,5)\quad \forall k\in [1,{{{{\mathrm{.}}}}}.,K]$$

4 $$\beta =\tau \tilde{\beta }$$

5 $${\tau }_{m,k}=\frac{\exp ({t}_{m,k})}{1+\exp ({t}_{m,k})}\forall m\in [1,\ldots ,M],\,\forall k\in [1,\ldots ,K]$$

6 $$\frac{{t}_{m,k}}{50} \sim {{{{{\rm{N}}}}}}(0,1)\,\forall m\in [1,\ldots ,M],\,\forall k\in [1,\ldots ,K]$$

7 $${\tilde{\beta }}_{m,k}={\sigma }^{2}{\gamma }_{m,k}\forall m\in [1,{{{{\mathrm{.}}}}}.,M],\,\forall k\in [1,{{{{\mathrm{.}}}}}.,K]$$

8 $${\sigma }_{m}^{2} \sim {{{{{\rm{HC}}}}}}(1)\forall m\in [1,{{{{\mathrm{.}}}}}.,M]$$

9 $${\gamma }_{m,k} \sim {{{{{\rm{N}}}}}}(0,1)\forall m\in [1,{{{{\mathrm{.}}}}}.,M],\forall k\in [1,{{{{\mathrm{.}}}}}.,K]$$

with *N* describing a Normal and HC a Half-Cauchy distribution following Polson et al.’s suggesting of hyperpriors for global scale parameters^31^.

To prevent identifiability issues of the covariate parameters, we reparametrize the model and choose one cell type *k* as a reference, forcing its covariates ${\beta }_{k}=0$ as in Maier et al.^11^ (Methods—“Automatic reference selection”).

Parameter inference is performed via Hamiltonian Monte Carlo (HMC) sampling using ten leapfrog steps per iteration with automatic step size adjustment according to Betancourt et al.^32^. Per default 20,000 iterations are performed with 5,000 iterations used as burn-in. The parameters ${\alpha }_{k},{\gamma }_{m,k}$ are randomly initiated by drawing from standard normal priors. ${t}_{m,k}$ is always initialized with 0 to ensure unbiased model selection, while ${\sigma }^{2}$ is initialized with 1. If the data contains entries that are zero, a pseudocount of 0.5 is added to these zero counts to reduce numerical instabilities.

After parameter inference, we calculate the inclusion probability ${P}_{inc}({\beta }_{k,m})$ of the covariates as follows:

10 $$\begin{array}{c}P({\beta }_{m,k})=\frac{1}{H}\mathop{\sum }\limits_{h=1}^{H}{{{\mathbb{I}}}}(|{\beta }_{m,k,h}|\ge {10}^{-3})\end{array}$$

with H the number of HMC iterations and ${{{\mathbb{I}}}}$ the indicator function. To identify credibly associated covariates, we compare the calculated inclusion probabilities with a decision threshold *c*, which is determined *a posteriori* to control for the false discovery rate (Methods—“Spike-and-slab threshold determination”). For credible effects, we report the effect parameter ${\beta }_{m,k}$ as the mean over all MCMC samples where ${\beta }_{m,k}$ was nonzero.

### Spike-and-slab threshold determination

To identify statistically credible effects, scCODA compares the posterior inclusion probability to a threshold *c*. As noted previously^33,34^, Bayesian variable selection methods must control for multiplicity, to avoid an inflated number of false-positive associations. To this end, we use a direct posterior probability approach^9,35^ to estimate the false discovery rate for a threshold value *c*.

By taking the posterior inclusion probability $P({\beta }_{m,k})$ as an approximation for the certainty of a credible effect for each ${\beta }_{m,k}$, its complementary $1-P({\beta }_{m,k})$ approximates the probability of a type I error. For a threshold *c*, we now rank all ${\beta }_{m,k}$ by their type I error probability and obtain a set of credible effects $J(c)=\{{\beta }_{m,k}|1-P({\beta }_{m,k})\le c\}.$Then, the approximate false discovery rate for the threshold is

11 $$\widehat{{{{{{\rm{FDR}}}}}}}(c)=\frac{{\sum }_{{\beta }_{m,k}\in J(c)}1-P({\beta }_{m,k})}{|J(c)|}.$$

For a desired false discovery rate *α*, we now set the optimal threshold $c^{\prime}$ to include as many effects as possible, without the approximate FDR exceeding *α*:

12 $$\begin{array}{c}c^{\prime} =\mathop{\min }\limits_{0 < c < 1;\widehat{{{{{{\rm{FDR}}}}}}}(c) < \alpha }c\end{array}$$

Finally, $J(c^{\prime} )$ is the set of credible effects that is reported by scCODA.

### Automatic reference selection

The compositional nature of scRNA-seq population data only allows statements about changes in abundance with respect to a reference group^5,8,11^. One way of defining such a reference is by selecting one cell type and interpreting changes to the other cell types with respect to this reference type. scCODA achieves this by forcing all effects on the reference cell type to be zero. The reference should therefore be set to a cell type that is known to be unaffected by the covariates.

However, such a cell type might not be known a priori. To alleviate this problem, scCODA offers an automatic reference selection that aims at selecting a cell type that is mostly unchanged in relative abundance, implying that the abundance of the reference cell type is stable over all samples. This is achieved by selecting the cell type that has the least dispersion of relative abundance over all samples, while being present in at least a fraction *t* of the samples:

13 $$\begin{array}{c}{K}_{ref}={{{{{\rm{argmi}}}}}}{{{{{{\rm{n}}}}}}}_{k\in \{1\ldots K\}}{{{{{\rm{Disp}}}}}}({Y}_{.,k}^{{\prime} })\; {{{{{\rm{s.t.}}}}}}\; \frac{|\{n:{Y}_{n,k} > 0\}|}{N}\ge t.\end{array}$$

Here, $Y^{\prime}$ is the relative abundance of cell counts. The additional condition on the reference cell type occurring in almost every sample is necessary to prevent very rare cell types from being selected, where small random changes in cell counts have a large impact on the relative abundance. Therefore, we recommend setting $t=0.95$, meaning that the reference cell type has to be present in at least 95% of samples. If no such cell type exists, this constraint can be relaxed by lowering *t*.

We now show how the choice of the reference cell type can influence the results of scCODA. As an example, we use the ulcerative colitis Lamina propria data from Smillie et al.^1^, comparing healthy and non-inflamed samples. We applied scCODA to this data 37 times, setting each cell type as the reference once (FDR level 0.05). Supplementary Fig. 8 shows the credible effects and effect size for each reference. For reference cell types that were mostly unchanged, i.e., were almost never found to be differentially abundant in the other runs, the found credible effects are largely consistent. On the other hand, cell types that were assigned a large negative effect (CD4+ activated Fos-lo, plasma cells) found significantly less credible effects when used as the reference, as the null level for the change is already negative. Taking epithelial cells, the only increasing cell type, as the reference led to the largest number of credible negative effects in other cell types. This shows that the reference cell type can have a large impact on the results of scCODA and should therefore be chosen with care.

### Credible intervals

To measure the certainty of scCODA’s credible effects, we calculate high-density intervals^36^ for each effect parameter ${\beta }_{m,k}$. Due to the spike-and-slab prior formulation, posterior samples of *β* are naturally zero-inflated, with the extent depending on each effect’s inclusion probability.

To counteract this bias, we, therefore, report credible intervals under the assumption that the effect in question is included in the model by calculating the high-density interval for each effect only across MCMC samples where the corresponding spike-and-slab variable was not 0:

14 $$\widehat{HDI}({\beta }_{m,k})=HDI({\beta }_{m,k}|{\tau }_{m,k} \, > \, 0).$$

Supplementary Fig. 11 shows how excluding the non-credible samples changes the 95% HDI for the example of healthy vs. non-inflamed samples of ulcerative colitis from the Lamina Propria^1^. While excluding the zero samples from the HDI calculation influences the HDI of most cell types only marginally, some high-density intervals become slightly wider (CD69- mast cells) or shift away from zero (cycling B cells). The average width of 95% HDIs increases only slightly from 0.92 to 0.97, though. Note that generally Bayesian high-density intervals are relatively large due to the MCMC sampling uncertainty.

### Simulation description

We carried out all benchmark studies by repeatedly generating compositional datasets $y\in {N}^{({n}_{0}+{n}_{1})xK}$ that have similar properties as the data from scRNA-seq experiments. For all synthetic datasets, we assumed a case-control setup with ${n}_{0}$ and ${n}_{1}$ samples in the two groups and *K* cell types, as well as a constant number of cells $\bar{y}$ in each sample.

We generated the synthetic datasets rowwise, with each row a sample of a Multinomial (MN) distribution ${{{{{{\boldsymbol{y}}}}}}}_{{{{{{\boldsymbol{i}}}}}}}={{{{{\rm{MN}}}}}}({{{{{\boldsymbol{\alpha }}}}}},\bar{y})$, and the probability vector ${{{{{\boldsymbol{\alpha }}}}}}$ a softmax transformation of a multivariate normal (MVN) sample: ${{{{{\boldsymbol{\alpha }}}}}}={{{{{\rm{softmax}}}}}}({{{{{\rm{MVN}}}}}}({{{{{\boldsymbol{\mu }}}}}},\,\Sigma ))$. We always used a covariance matrix of $\Sigma =0.05\, I{d}_{K}$, which mimics the variances observed in the experimental data of Haber et al., while assuming no correlation between the cell types besides the compositional effects^6^.

In the power, heterogeneous response, and runtime analysis benchmarks, the mean vector *μ* for each sample was calculated from the mean abundance of the first cell type in control samples (no effect) ${\mu }_{0}$, and the mean change in abundance of the first cell type between the two groups $\mu ^{\prime}$. All other cell types were modeled to be equally abundant, leading to ${{{{{\boldsymbol{\mu }}}}}}=\,\log ({\mu }_{0},\frac{\bar{y}-{\mu }_{0}}{K-1},\frac{\bar{y}-{\mu }_{0}}{K-1},\ldots )$ for control samples, and ${{{{{\boldsymbol{\mu }}}}}}=\,\log ({\mu }_{0}+\mu ^{\prime} ,\frac{\bar{y}-({\mu }_{0}+\mu ^{\prime} )}{K-1},\frac{\bar{y}-({\mu }_{0}+\mu ^{\prime} )}{K-1},\ldots )$ for samples in the other group.

For the model comparison benchmark, we also included effects on two different cell types. For this, we assumed ${{{{{\boldsymbol{\mu }}}}}}={{{{{\mathrm{log}}}}}}(1000,1000,\ldots ,1000)$ for all control samples, and an increase of ${{{{{\boldsymbol{\mu }}}}}}^{\prime} =({\mu }_{1}^{{\prime} },{\mu }_{2}^{{\prime} })$ on the first two cell types, leading to ${{{{{\boldsymbol{\mu }}}}}}={{{{{\mathrm{log}}}}}}(1000+{\mu }_{1}^{{\prime} },1000+{\mu }_{2}^{{\prime} },\frac{K\cdot 1000\mbox{-}(2000+{\mu }_{1}^{{\prime} }+{\mu }_{2}^{{\prime} })}{K-2})$.

For all benchmark studies, we defined sets of values for all parameters mentioned above and generated *r* datasets for every possible parameter combination. We then applied scCODA with the last cell type chosen as reference to each synthetic dataset. For the model comparison benchmark (Methods—“Model comparison”), we analyzed the results at FDR levels of 0.05 and 0.2. The overall benchmark (Methods—“Power analysis”), heterogeneous response benchmark (Methods—“Analysis of heterogeneous response groups”) and runtime analysis (Methods—“Runtime analysis”) were carried out with an expected FDR level of 0.05.

The sets of generation parameters were as follows:

- Model comparison (Fig. 2, Methods—“Model comparison”):
  $$K=\{5,10,15\};$$
  $${n}_{0}={n}_{1}=\{1,2,3,4,5\}({{{{{\mathrm{only}}}}}}\,{{{{{\mathrm{balanced}}}}}}\,{{{{{\mathrm{setups}}}}}}-{n}_{0}={n}_{1});$$
  $$\bar{y}=K\cdot 1000;$$
  $${\mu }_{0}=1000;$$
  $${{{{{{\boldsymbol{\mu }}}}}}}^{\prime}=(0,500);(0,1000);(0,2000);(500,1000);(500,2000);(1000,2000);$$
  $$r=20;$$
- Power analysis (Supplementary Fig. 4, Methods—“Power analysis”):
  $$K=5;$$
  $${n}_{0}={n}_{1}=\{1,2,{{{{\mathrm{..}}}}}.,10\}({{{{{\mathrm{also}}}}}}\,{{{{{\mathrm{imbalanced}}}}}}\,{{{{{\mathrm{setups}}}}}});$$
  $$\bar{y}=5000;$$
  $${\mu }_{0}=\{20,30,50,75,115,180,280,430,667,1000\};$$
  $${{{{{\boldsymbol{\mu }}}}}}^{\prime} =\{10,20,30,40,50,60,70,80,90,100,200,400,600,800,1000\};$$
  $$r=10;$$
- Heterogeneous response groups (Supplementary Fig. 9, Methods—“Analysis of heterogeneous response groups”)**:**
  $$K=5;$$
  $${n}_{0}={n}_{1}=20;$$
  $$\bar{y}=5000;$$
  $${\mu }_{0}=\{1,100,1000\};$$
  $${{{{{\boldsymbol{\mu }}}}}}^{\prime} =\{500,1000,2000\};$$
  $$r=20;$$
- Runtime analysis (Supplementary Fig. 12, Methods—“Runtime analysis”)**:**

$$K=\{5,10,15,\ldots 50\};$$

$${n}_{0}={n}_{1}=\{5,10,15,20\}({{{{{\mathrm{only}}}}}}\,{{{{{\mathrm{balanced}}}}}}\,{{{{{\mathrm{setups}}}}}}-{n}_{0}={n}_{1});$$

$$\bar{y}=100,000;$$

$${\mu }_{0}=\frac{1}{K};$$

$${{{{{\boldsymbol{\mu }}}}}}^{\prime} =\{1\};$$

$$r=20;$$

### Power analysis

To be able to estimate the required sample sizes for an intended MCC, we fitted a quasibinomial regression model with log-linker function using log sample size, log absolute change in cell count, log-fold change, and all pairwise interactions using the simulation results at different fixed FDR levels (FDR=[0.05, 0.1, 0.2]; Methods—“Simulation description”).

We performed a backward model selection with repeated tenfold cross-validation to reduce the feature set. The final model consisted of log sample size, log-fold change, log-fold change, and the interaction effects between log total sample size and log absolute cell count change as well as log absolute cell count and log-fold change, which was expected given that we observed an interaction effect between these two variables in the raw benchmark results (Supplementary Fig. 4d–f).

With the fitted model, we inverse estimated the required log total sample size ${x}_{ss}$ with fixed TPR ${y}_{tpr}$, log-fold change ${x}_{fc}$, and log absolute cell count change ${x}_{cc}$ as:

15 $${x}_{ss}=\frac{-(\alpha +{\beta }_{fc}{x}_{fc}+{\beta }_{fc,cc}{x}_{fc}{x}_{cc}-{y}_{tpr})}{({\beta }_{ss}+{\beta }_{ss,cc}{x}_{cc})}$$

With this formula, we estimated the sample size for a fixed power of 0.8 across changing log-fold changes between [0.01, 5] and the fraction of cell-type sizes to total cell counts between [0.01, 0.2] for the same fixed FDR levels.

### Analysis of publicly available datasets

#### Single-cell RNA-seq data of PBMCs from supercentenarians

We downloaded the processed single-cell RNA-seq count matrices comprising PBMCs of seven supercentenarians and five younger controls from http://gerg.gsc.riken.jp/SC2018/. Read counts were log-transformed and PCA embedded using the first 50 PCs. Leiden clustering was used to cluster cells into major groups. Following the described analysis in Hashimoto et al.^3^, we annotated the major cell types including T cells characterized by *CD3* and T-cell receptor (*TRAC*) expression, B cells characterized by *MS4A1* (*CD20*) and *CD19* expression, natural killer cells characterized by *KLRF1* expression, monocytes characterized by *CD14* and *FCGR3A* (*CD16*) expression, respectively, and erythrocytes characterized by *HBA1* expression, and determined their cell counts per sample (Supplementary Fig. 5). All analysis steps were carried out using Scanpy v.1.5.1.

#### Single-cell RNA-seq data of microglia in Alzheimer’s disease (AD) mouse model

We downloaded the raw single-cell RNA-seq count matrices (deposited at GEO, accession code GSE98969) comprising immune cells isolated from the mouse brain in wild-type (WT) and AD mice^19^. The complete dataset with all samples consists of 37,248 cells. We filtered out ERCC spike-ins before computing the quality metrics of all cells. We then excluded 12,053 cells with less than 500 UMI counts and 11,065 genes, which were not expressed. We subsequently normalized by library size with target sum 10,000 counts (CPM normalization) and log+1 scaled. Following the analysis of Keren-Shaul et al.^19^, we selected the samples of six-month-old mice from AD and WT, which have not been sorted by brain region, resulting in 9,196 cells. It must be noted that Keren-Shaul et al. reported 8,016 cells when they first annotated immune cells in 6-month-old mice (see Fig. 1 in Keren-Shaul et al.). We evaluated batch effects based on the clustering results and visual inspection of the UMAP plots, where none of the samples clustered separately in any of the clusters, which is, in this case, sufficient to obtain cell types. We clustered the data using Louvain clustering with resolution 1 and annotated cell types using the previously reported marker genes as microglia 1 (*CTSD, CD9, HEXB, CST3*), microglia 2–3 (*LPL, CST*), granulocytes (*CAMP, S100a9*), T/NK cells (*S100a4, NKG7, Trbc2*), B cells (*RAG1, CD79b, CD74*), monocytes (*S100a4, CD74*), perivascular macrophages (*CD74, CD163, MRC1*) (see Supplementary Fig. 6). We subsequently sub-clustered the microglia population into three clusters, assigning the labels microglia 1, 2, and 3, respectively. Similar to Keren-Shaul et al., we assigned the region-sorted samples of AD and WT mouse model (*n* = 2 per region) with a k-nearest neighbor classifier (*k* = 30). We then evaluated the number of unassigned cells, performed another round of Louvain clustering, and assigned the remaining cells based on the majority vote for the clustering result, i.e., when unassigned cells clustered predominantly with microglia 1, they were all assigned to microglia 1. The obtained proportions of microglia subpopulations are in accordance with the previously reported proportions. All analysis steps were carried out using Scanpy v.1.5.1.

#### Single-cell RNA-seq data of ulcerative colitis in human donors

We used the annotated single-cell RNA-seq data of the colon epithelium from 12 healthy donors and 18 patients with chronic inflammation^1^. From healthy donors, samples from two adjacent locations were taken. From patients, biopsies from inflamed and adjacent normal tissue (“non-inflamed”) were taken. Further, the biopsies were separated by enzymatic digestion into the epithelium (“Epi”) and the lamina propria (“LP”) before single-cell RNA-sequencing. The study comprises a total of 365,492 transcriptomes from 133 samples. The data were downloaded from Single Cell Portal (accession ID SCP259). The analysis code and description were provided at https://github.com/cssmillie/ulcerative_colitis.

The original study annotated all cell types together, resulting in 51 different cell types. However, some cell types that are originally located in the LP have been found in the epithelial samples and vice versa. For the differential composition analysis of the Epi and LP, we considered the nonepithelial and epithelial cell types, respectively, as one group. Therefore, we tested the changes in 16 cell types in the Epi and 37 cell types in LP. In addition, we reanalyzed the data using the Dirichlet regression model as in Smillie et al.^1^ (with R package DirichletReg v.0.7-0 in R v.3.5.2). Importantly, we realized that Smillie et al. summed up the counts of the same replicates (as described in the analysis scripts in https://github.com/cssmillie/ulcerative_colitis), while we consider every replicate as an independent. Overall, we have data from 29 donors (61 samples, where 24 healthy, 21 non-inflamed, 16 inflamed) in Epi and data from 30 donors (72 samples, where 24 each healthy, non-inflamed, and inflamed, respectively) in LP. Specifically, we tested chain lengths of 20,000, 40,000, 80,000, and 150,000 iterations with a burn-in of 10,000 in the Epi case, while we tested chain lengths of 200,000, 400,000, and 800,000 iterations with a burn-in of 10,000 in the LP case due to the larger number of cell types in LP compared to Epi.

#### Single-cell RNA-seq data of bronchoalveolar immune cells in patients with COVID-19

We used the annotated single-cell RNA-seq data of the bronchoalveolar lavage fluid cells from three patients with moderate COVID-19 progression, six patients with severe COVID-19 progression, four healthy donors, and a publicly available sample^4^. The cell-type annotations of all samples were provided at https://github.com/zhangzlab/covid_balf.

#### Single-cell RNA-seq data of small intestinal epithelial cells infected with different bacteria

Annotated single-cell transcriptomics data of epithelial cells from the small intestine of mice infected with three different bacterial conditions were downloaded from Single Cell Portal (accession ID SCP44). The data consisted of a control group of four mice (3,240 cells total) and three groups of two mice each, measured after 2 days for *Salmonella* (1,770 cells total), as well as three (2,121 cells total) and ten days (2,711 cells total) after *H. polygyrus* infection, respectively.

### Model comparison

We compared scCODA’s ability to correctly identify significant compositional changes in a setting typical for single-cell experiments to other methods recently used in scRNA-seq analysis and approaches from the field of microbial population analysis. We applied all methods to each of the 5,000 datasets generated for the comparison analysis (Methods—“Simulation description”) and recorded which of the cell types each method found to be differentially abundant between the two groups. We then compared these results to the ground truth assumption from the data-generation process via binary classification metrics (credible vs. non-credible changes). We chose Matthews’ correlation coefficient as our primary metric, as it best accounts for the numerical imbalance between the two groups. Details on the individual differential abundance testing methods can be found in Supplementary Table 2. We also investigated the False discovery rate and sensitivity (true positive rate) for each method for a more detailed performance analysis.

Furthermore, we performed sensitivity analysis via the receiver-operating characteristic and precision-recall curve. The different methods use different metrics (e.g., *P* values) that can be thresholded to obtain the sensitivity curves. The thresholding metric, AUC score, and average precision score for each method are listed in Supplementary Table 1.

### Analysis of heterogeneous response groups

In certain cases, only a fraction of the samples in a treatment group show a response to the stimulus. To quantify the sensitivity of scCODA in such scenarios, we conducted another benchmark study. We simulated datasets as before, assuming that either a rare or an abundant cell type was increasing by a significant margin in the treatment group (Methods—“Simulation description”). To mimic a partial response to the covariate, we defined treatment groups where the affected cell type was increased in (5%, 10%, … 100%) of the samples, while the rest of the samples followed the distribution of the control group.

Independent of the abundance of a cell type, scCODA detected the effects only if a relatively large share of the samples was responsive to the condition. For abundant cell types (base count ${\mu }_{0} = 100\, {{{\mbox{or}}}}\,1000$), a response share of about 40% was enough to achieve reliable detection, while for very rare cell types (base count ${\mu }_{0}=1$), more than half of the samples needed to show a response. If the share of responding samples was 70% or higher, scCODA reliably detected the effects (Supplementary Fig. 9).

We therefore conclude that scCODA is robust to small amounts of non-responding samples within a condition. However, scCODA does not detect compositional changes that only manifest in a minority share within a condition. In that case, the changes will be considered as outliers rather than credible effects.

### Runtime analysis

To benchmark the execution time and scalability of scCODA with the size of the data, we generated a collection of 800 datasets with an increasing number of cell types and samples (Methods—“Simulation description”). The generation parameters were chosen such that the typical dimensions of scRNA-seq datasets are covered by the benchmark.

scCODA uses HMC sampling for parameter inference. Therefore, the most important factor in runtime is the duration of one HMC sampling step. To isolate the HMC sampling process from the model initialization and post-sampling analysis steps, we applied scCODA twice to each dataset, sampling chains of length 1,000 and 2,000, respectively. We measured the execution time for both instances and divided the time difference by 1,000—the difference in chain length—to gain an estimate for the execution time per sampling iteration (Supplementary Fig. 12). All operations were executed on an Intel(R) Xeon(R) Gold 6126 processor. The memory consumption of a single run of scCODA in default settings should not exceed 2 GB.

For five cell types, datasets of all tested sample sizes require about 0.0025 s per HMC iteration on average. The time per iteration increased linearly with the number of cell types for all sample sizes. This effect is more pronounced for larger sample sizes, with 40 total samples (20 per group) and 50 cell types requiring the longest average time per step of about 0.0035 s, while the average runtime per step for datasets with five samples was always below 0.0027 s. Thus, running scCODA with the default number of 20,000 HMC iterations on any dataset of typical size should produce results within a few minutes.

### Reporting summary

Further information on research design is available in the Nature Research Reporting Summary linked to this article.

## Supplementary information

Supplementary Information

Description of Additional Supplementary Files

Supplementary Data 1

Supplementary Data 2

Supplementary Data 3

Supplementary Data 4

Supplementary Data 5

Reporting Summary

## Data Availability

The synthetic benchmark datasets and results have been deposited on Zenodo at 10.5281/zenodo.4305907. The single-cell datasets can be found in their respective public repositories. The supercentenarians PBMC dataset by Hashimoto et al. can be found at http://gerg.gsc.riken.jp/SC2018, while the Alzheimer’s mouse microglia dataset by Keren-Shaul et al. can be accessed at GEO under GSE98969. The single-cell ulcerative colitis dataset by Smillie et al. can be downloaded from the Single-Cell Portal (Accession ID SCP259) and its accompanying analysis code and description from https://github.com/cssmillie/ulcerative_colitis. The processed single-cell data of bronchoalveolar immune cells in patients with COVID-19 by Liao et al. is publicly available at https://github.com/zhangzlab/covid_balf. The single-cell data of small intestinal epithelial cells infected with different bacteria is available from Single Cell Portal (accession ID SCP44).

## Code availability

The method has been implemented in Python 3.8 using Tensorflow = 2.3.2^37^, Tensorflow-Probability = 0.11^38^, ArviZ >= 0.10^39^, numpy >= 1.19, and Scanpy >= 1.5^12^. The Power Analysis was performed using caret package^40^ (R 4.1). Source code has been deposited on Github at https://github.com/theislab/sccoda^41^. All code to reproduce the presented analyses can be found on Github at https://github.com/theislab/scCODA_reproducibility^42^. All tested methods have been integrated into a unifying Python API that can directly interact with Scanpy and Anndata.
