# Supplementary material for: scCODA is a Bayesian model for compositional single-cell data analysis
Source: Nat Commun. 2021 Nov 25;12:6876. doi: 10.1038/s41467-021-27150-6 (PMC8616929; doi:10.1038/s41467-021-27150-6)
Supplement: Supplementary file 1 — Supplementary Information [file 41467_2021_27150_MOESM1_ESM.pdf]

scCODA is a Bayesian model for compositional single-cell data analysis

Büttner M.[1+], Ostner J.[1,2+], Müller CL.[1,2,3*], Theis FJ.[1,4,5†], Schubert B.[1,4†*]

[1] Institute of Computational Biology, Helmholtz Zentrum München, German Research Center for Environmental Health, Neuherberg, Germany

[2] Department of Statistics, Ludwig-Maximilians-Universität München, München, Germany

[3] Center for Computational Mathematics, Flatiron Institute, New York, New York, USA

[4] Department of Mathematics, Technische Universität München, Garching bei München, Germany

[5] TUM School of Life Sciences Weihenstephan, Technical University of Munich, Freising, Germany

[+]These authors contributed equally.

[†]These authors jointly supervised this work.

*Correspondence: christian.mueller@stat.uni-muenchen.de; benjamin.schubert@helmholtz-muenchen.de

Supplementary Figures

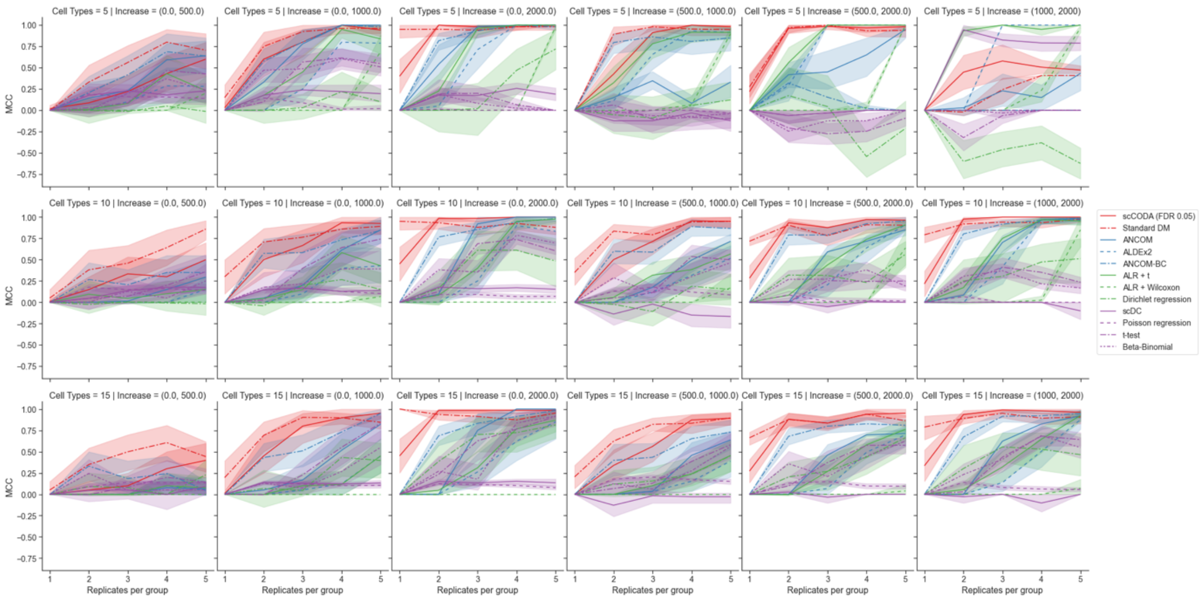

Supplementary Figure 1: Comparison of model performance by sample size measured by MCC, separated by number of cell types and effect size. The "Increase" value denotes the expected absolute change in the first two cell types between control and case samples. Error bands denote the 95% confidence interval around the mean.

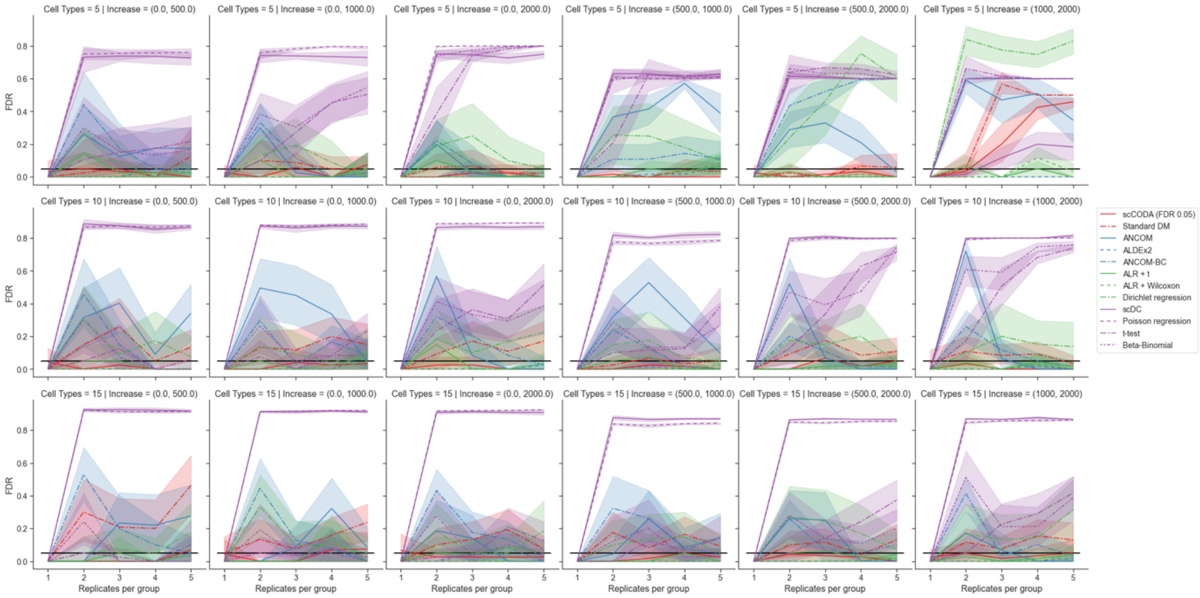

Supplementary Figure 2: Comparison of model precision by sample size measured by FDR, separated by number of cell types and effect size. The "Increase" value denotes the expected absolute change in the first two cell types between control and case samples. Error bands denote the 95% confidence interval around the mean. The nominal FDR level of 0.05 for all methods is indicated with a horizontal black line. The case of 5 cell types and an increase of (1000, 2000) shows much higher FDR values, because the reference cell type has an expected count of 0 in the case samples.

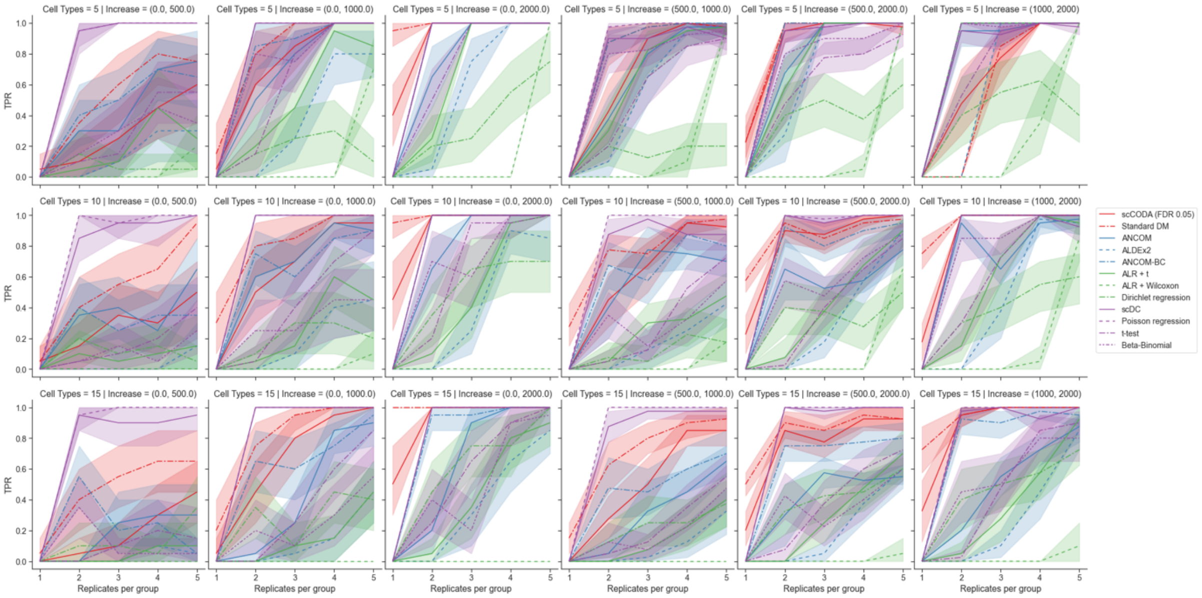

Supplementary Figure 3: Comparison of model sensitivity by sample size measured by TPR, separated by number of cell types and effect size. The "Increase" value denotes the expected absolute change in the first two cell types between control and case samples. Error bands denote the 95% confidence interval around the mean.

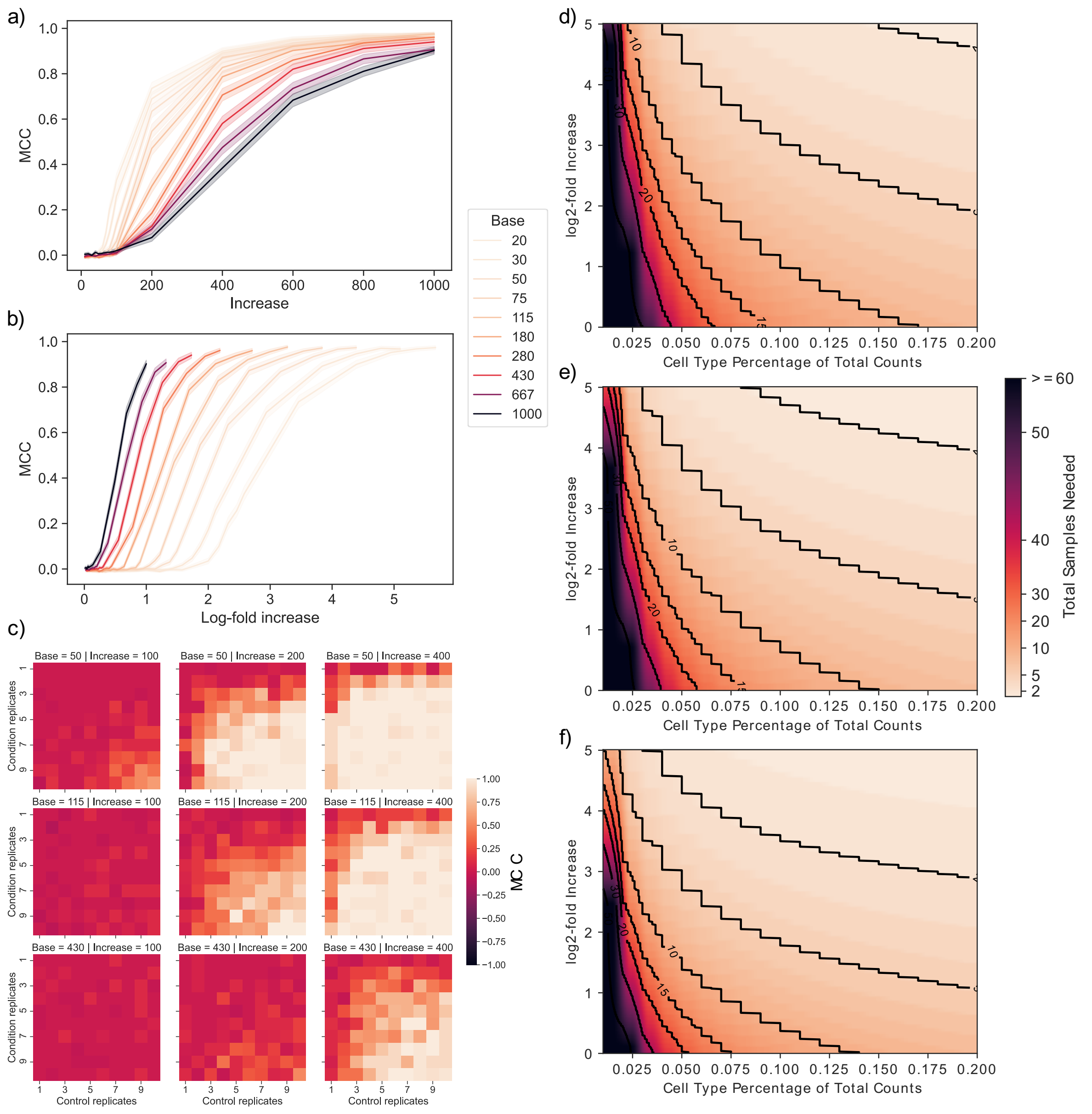

Supplementary Figure 4: Benchmark evaluation results for overall benchmark **(Methods - Simulation description)**. **(a)** The performance of scCODA (measured by MCC) depends on the amount of change in abundance. The “Base” value represents the mean cell count of the only differentially abundant cell type in the control group samples. For cell types with higher initial abundance, the absolute (count) change must be higher to reliably detect changes in abundance. Error bands denote the 95% confidence interval around the mean. **(b)** For cell types with higher initial abundance, scCODA can detect smaller relative (log2-fold) changes between the two groups. Error bands denote the 95% confidence interval around the mean. **(c)** Example

performance of scCODA depending on sample size of both groups. Less abundant cell types need a smaller absolute increase to be reliably detected as differentially abundant. The shaded areas in **(b-c)** represent 95% confidence intervals **(d-f)** Total samples per group needed to achieve an expected sensitivity of 0.8, depending on base value and increase of the differentially abundant cell type **(Methods - Power analysis)** for fixed FDR levels of **(d)** FDR=0.05, **(e)** FDR=0.1, and **(f)** FDR=0.2.

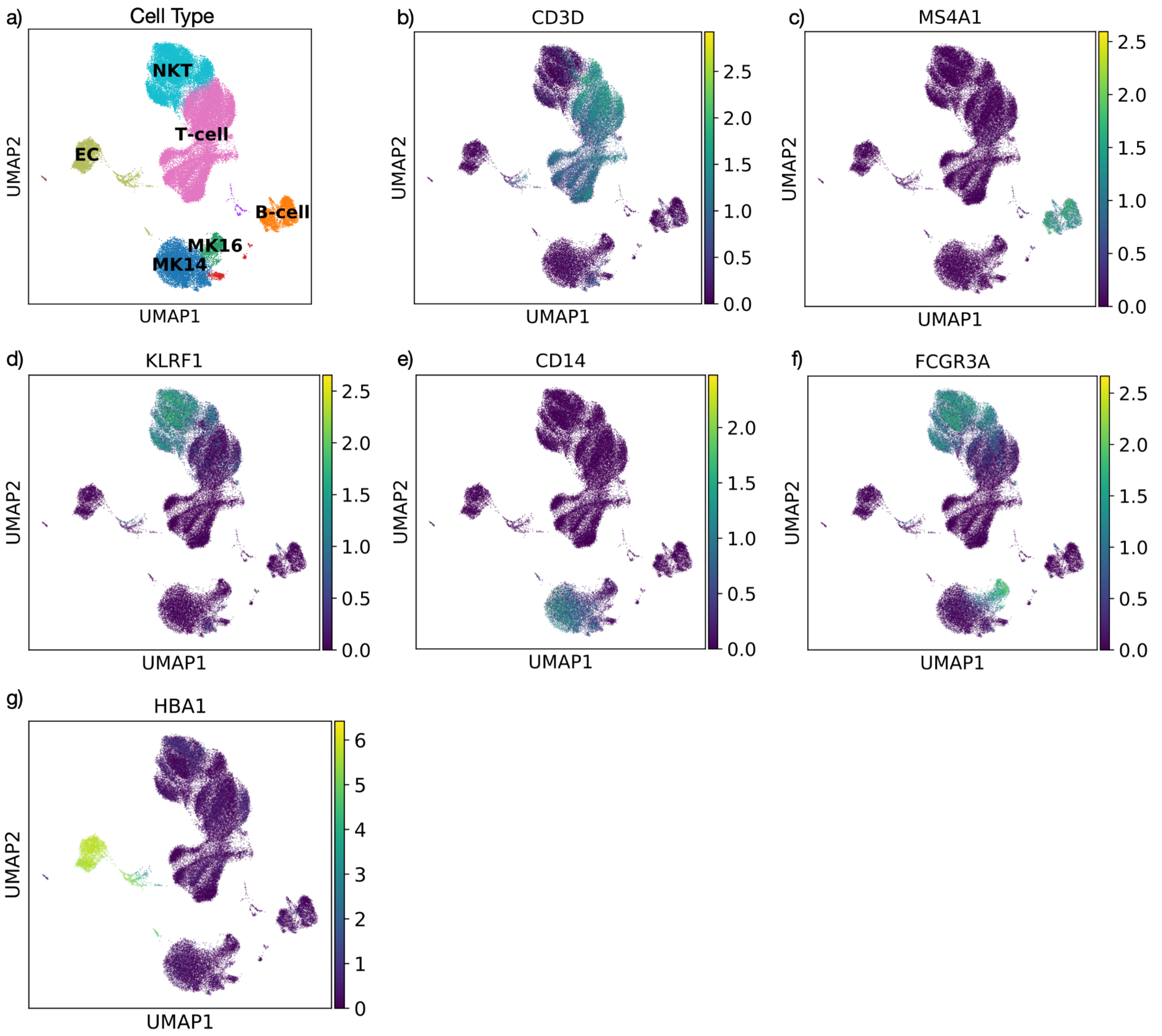

Supplementary Figure 5: Re-analysis of supercentenarian data of Hashimoto et al.[3]. **(a)** Final annotation of major cell types. **(b-g)** expression pattern of *CDR3* identifying T-cells, *MS4A1* identifying B-cells, KLRF*1* natural killer cells (NKT), *CD14* and *FCGR3A* (CD16) Monocyte subtypes (CD14+, CD16+, denoted as MK14 and MK16), and *HBA1* Erythrocytes (EC).

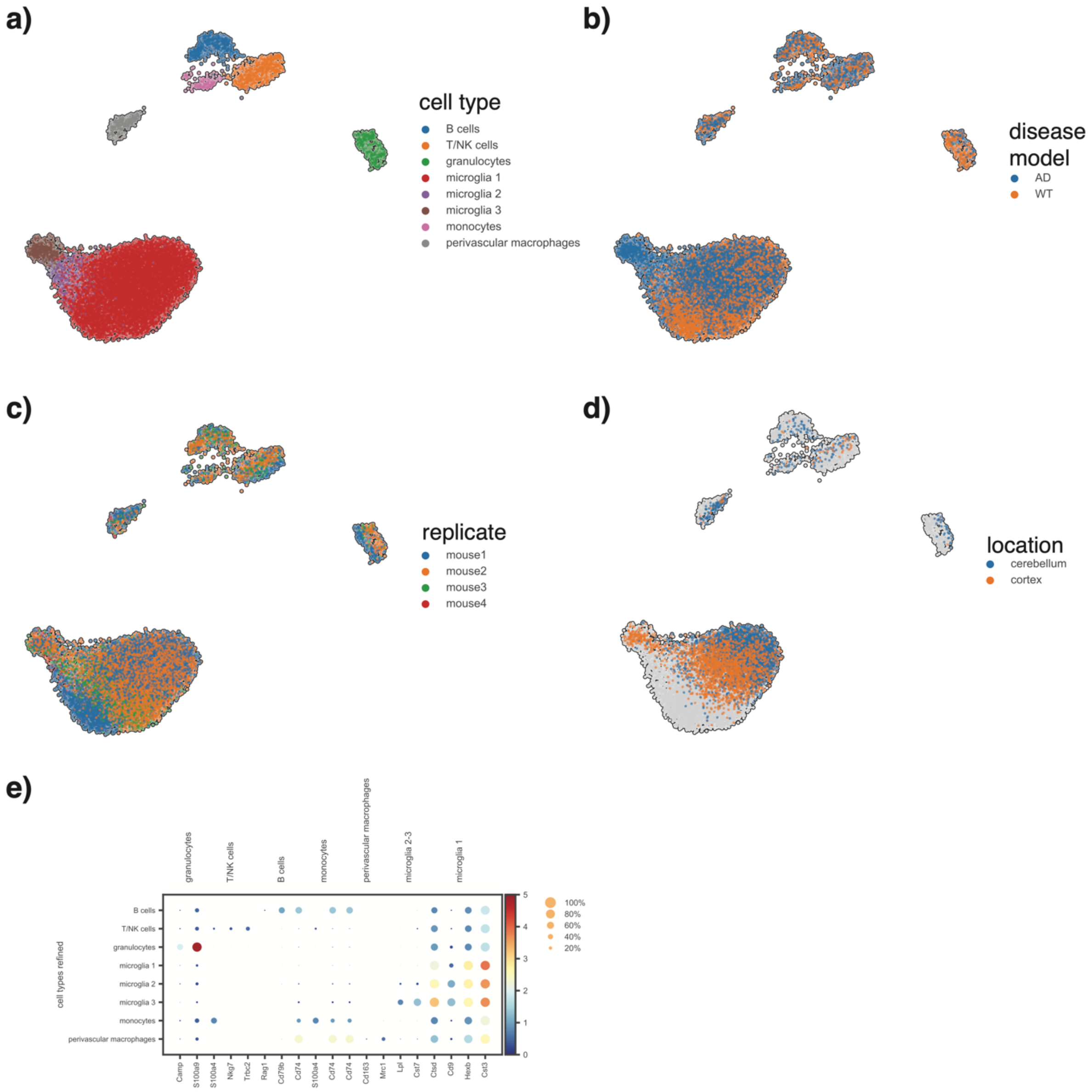

Supplementary Figure 6: Re-analysis of microglia data in Alzheimer's disease (AD) mouse model[19]. **(a)** Joint cell type annotation of cells. **(b)** Cell distribution in the both wild type (WT) and AD mouse models. **(c)** Distribution of cells from different replicates does not indicate strong batch effects. **(d)** Location of cells sorted from cortex and cerebellum. Location of grey cells was not reported. **(e)** Dot plot of marker gene expression of the annotated cell populations **(a)**.

a)

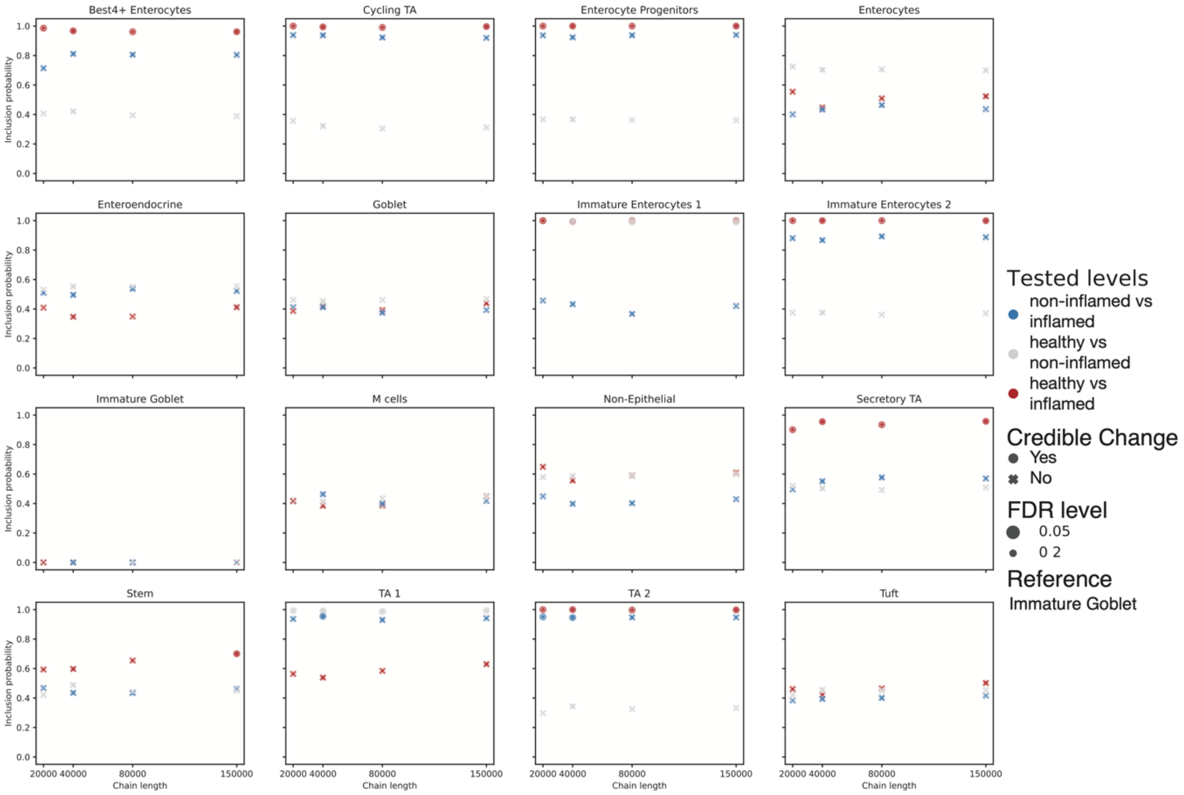
Best4+ Enterocytes
Cycling TA
Enterocyte Progenitors
Enterocytes
Enteroendocrine
Goblet
Immature Enterocytes 1
Immature Enterocytes 2
Immature Goblet
M cells
Non-Epithelial
Secretory TA
Stem
TA 1
TA 2
Tuft
Inclusion probability
Chain length
20000 40000 80000 150000
Tested levels
non-inflamed vs inflamed
healthy vs non-inflamed
healthy vs inflamed
Credible Change
Yes
No
FDR level
0.05
0 2
Reference
Immature Goblet

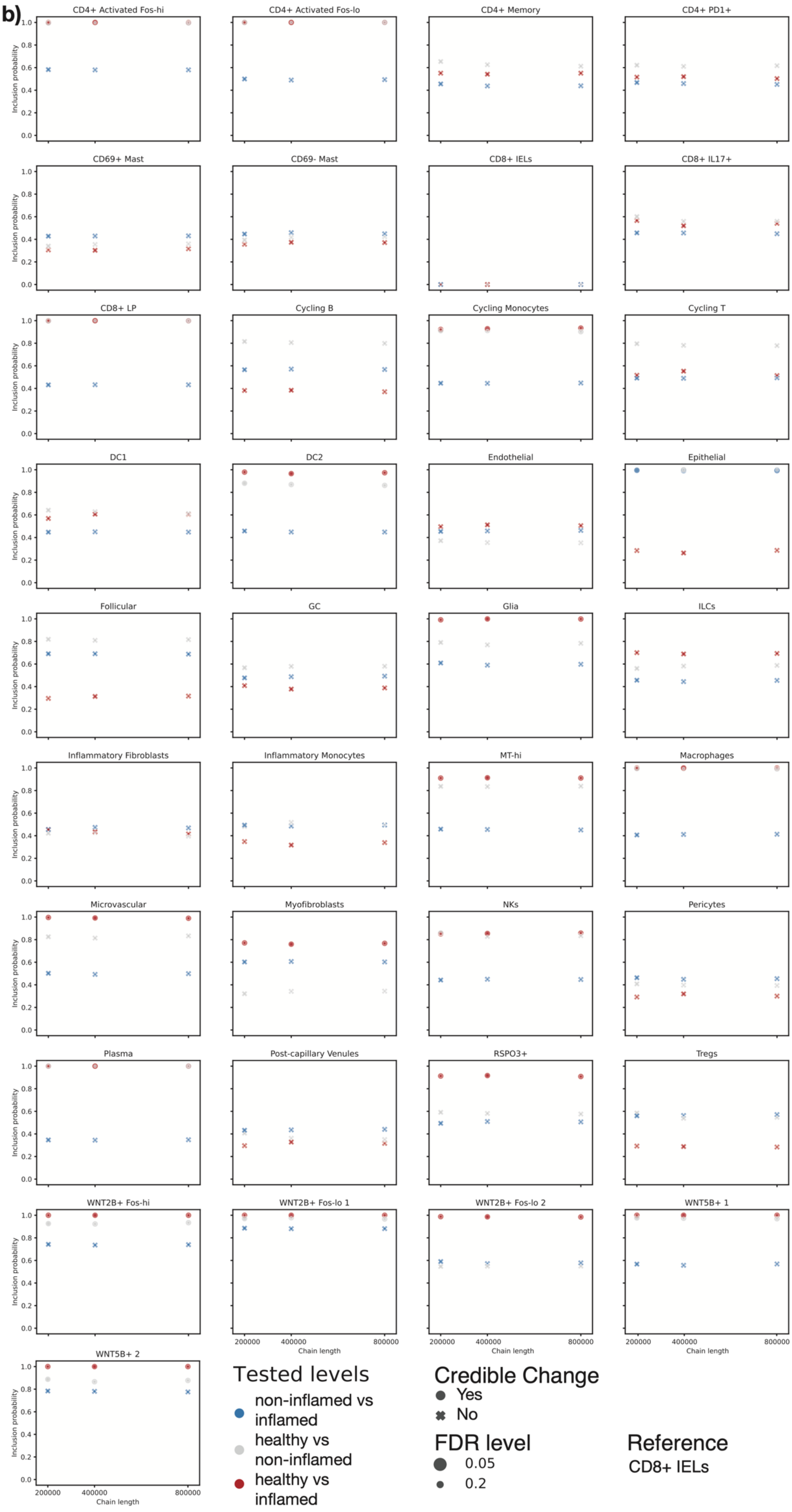

b)
CD4+ Activated Fos-hi
CD4+ Activated Fos-lo
CD4+ Memory
CD4+ PD1+
CD69+ Mast
CD69- Mast
CD8+ IELs
CD8+ IL17+
CD8+ LP
Cycling B
Cycling Monocytes
Cycling T
DC1
DC2
Endothelial
Epithelial
Follicular
GC
Glia
ILCs
Inflammatory Fibroblasts
Inflammatory Monocytes
MT-hi
Macrophages
Microvascular
Myofibroblasts
NKs
Pericytes
Plasma
Post-capillary Venules
RSPO3+
Tregs
WNT2B+ Fos-hi
WNT2B+ Fos-lo 1
WNT2B+ Fos-lo 2
WNT5B+ 1
WNT5B+ 2
Inclusion probability
Chain length
Tested levels
non-inflamed vs inflamed
healthy vs non-inflamed
healthy vs inflamed
Credible Change
Yes
No
FDR level
0.05
0.2
Reference
CD8+ IELs

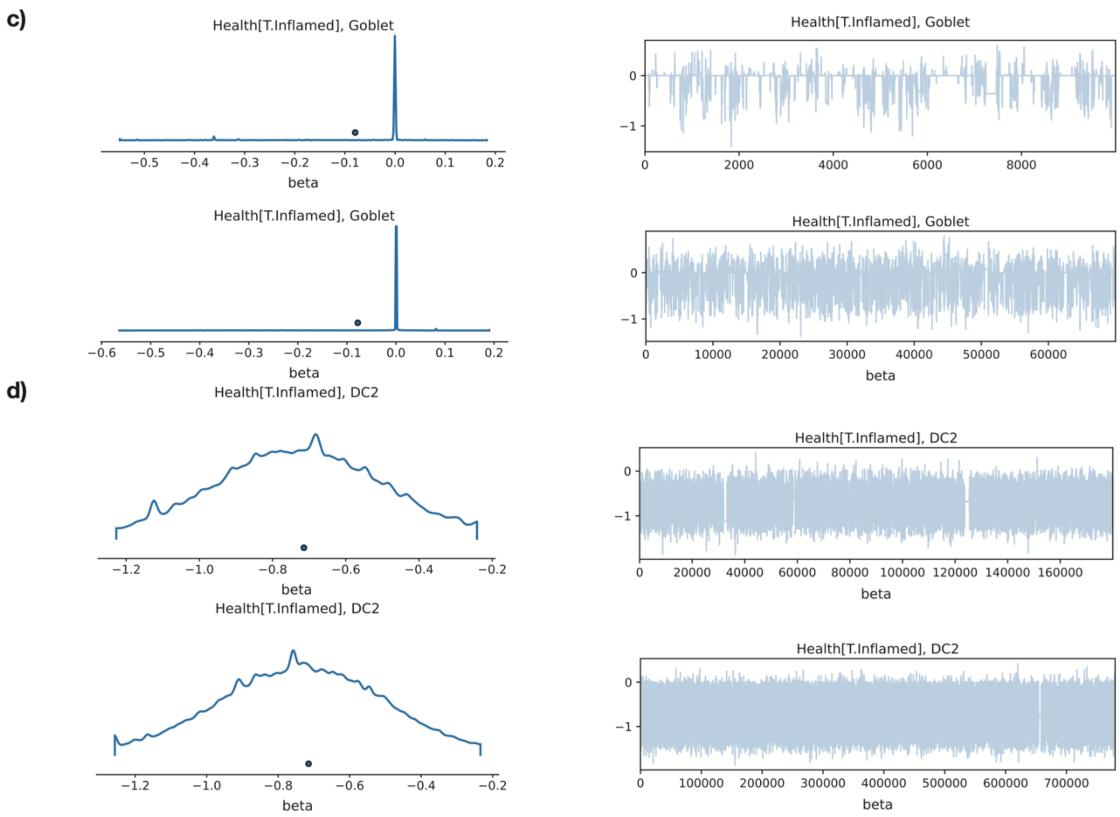

Supplementary Figure 7: Convergence of HMC sampling for many cell types (in data of Smillie et al.). **(a-b)** Inclusion probabilities for pairwise tests in the epithelium **(a)** and lamina propria **(b)** of healthy donors and patients of UC. Colors depict the tested levels; symbols depict the credibility of the changes. The effect of the reference is set to zero. **(c-d)** Density plots (left panels) and trace plots (right panels) of different chain lengths for the parameter inference in Goblet cells comparing healthy and inflamed samples (reference Immature Goblet) **(c)** and DC2 comparing healthy and inflamed samples (reference CD8+ IELs) **(d)**.

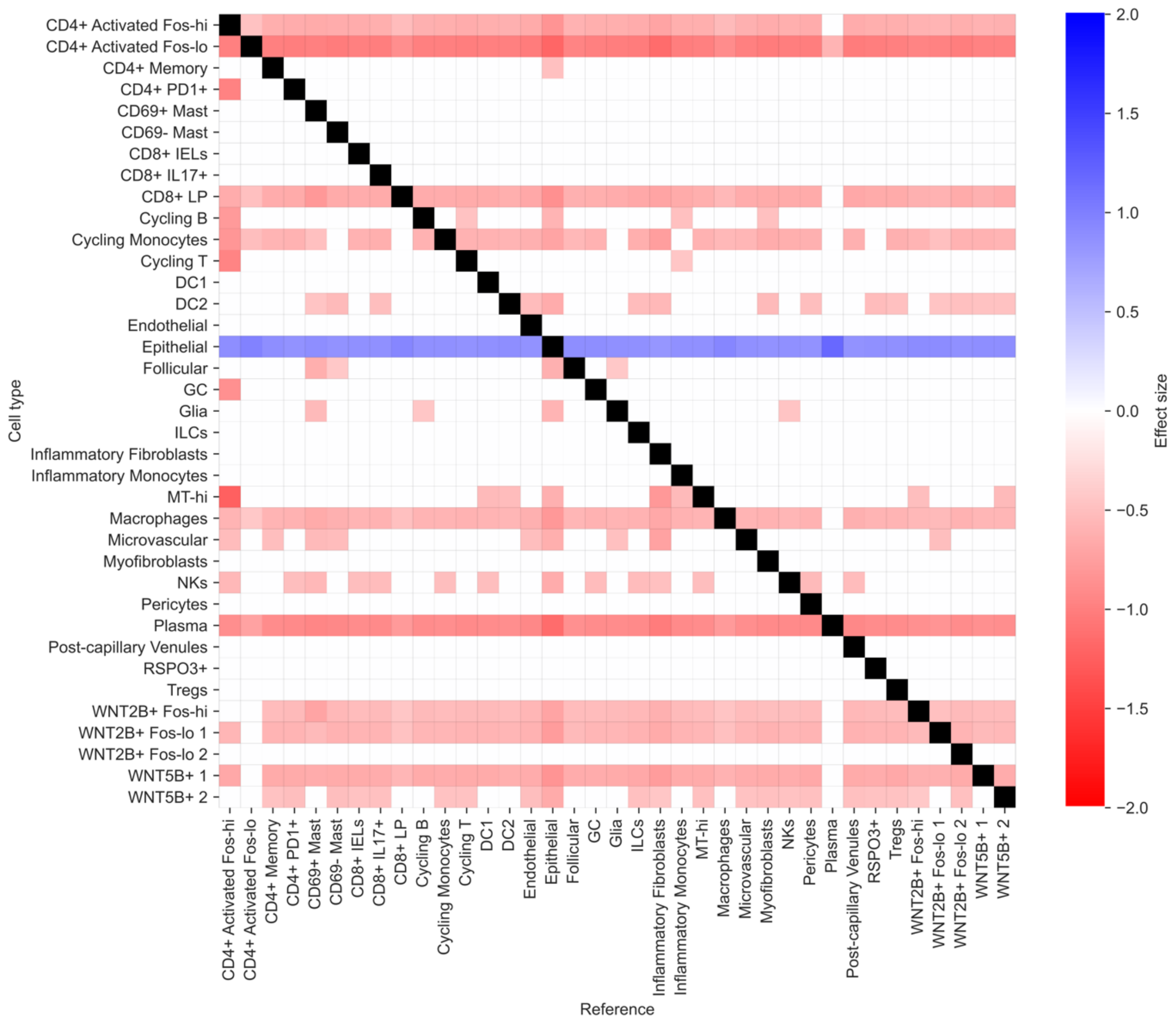

Supplementary Figure 8: Credible effects depend on the reference cell type in scCODA. Blue and red areas depict positive and negative credible effects, white areas show no credible effect. The reference cell type itself is colored black. Using a cell type as the reference that is often characterized with a large decrease (CD4+ Activated Fos-lo, Plasma cells) leads to less credible decreases being found. Using Epithelial cells, the only credibly increasing cell type, as the reference, leads to a larger number of negative effects. Data: Ulcerative colitis data from the Lamina propria (Healthy vs. non-inflamed)[1]

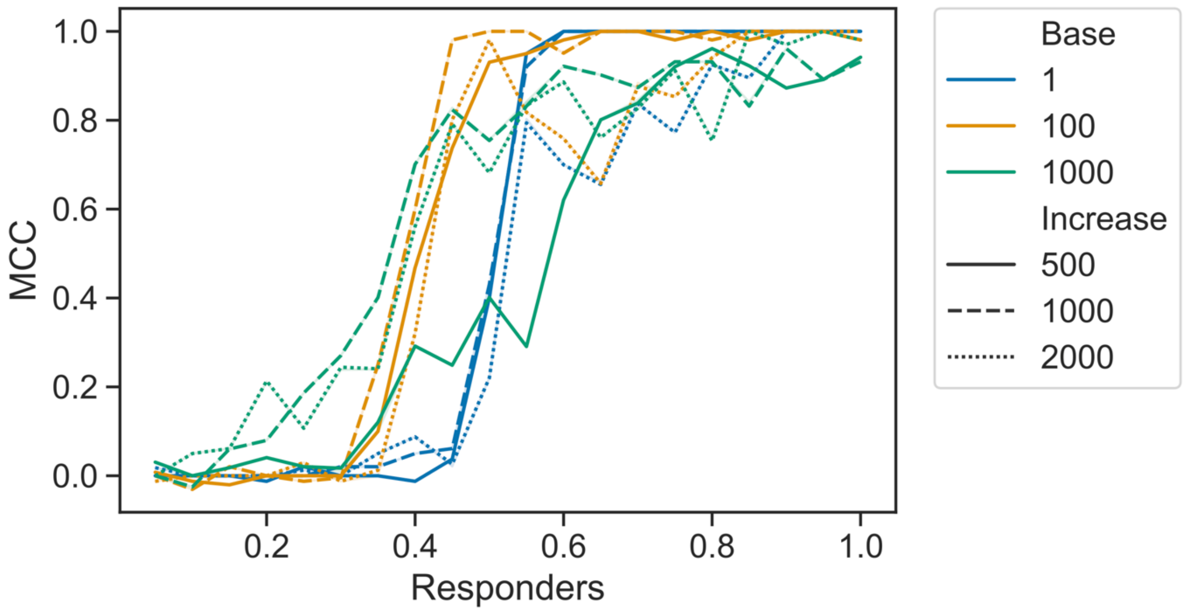

Supplementary Figure 9: Benchmarking results on response heterogeneity in a condition **(Methods - Analysis of heterogeneous response groups)**. The "Base" value indicates the mean count of the affected cell type in the control group, the "Increase" value represents the absolute increase between conditions. The x-axis shows the fraction of treatment samples that were simulated to respond to the condition. Only if more than half of the samples responded to the treatment, scCODA was able to reliably detect the effect, even in very rare cell types.

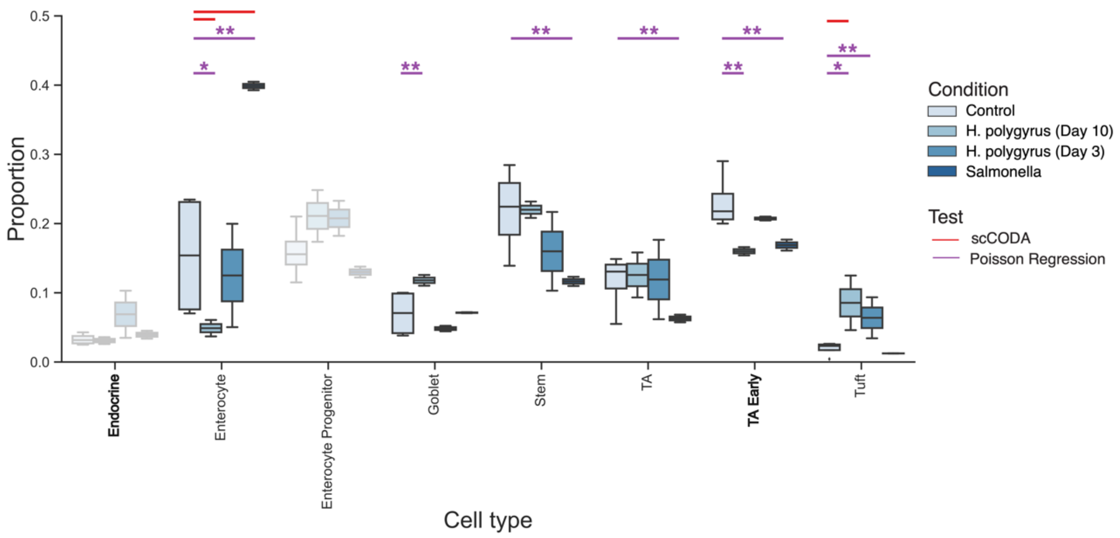

Supplementary Figure 10: Compositional analysis of Haber et al.[6] on the response to pathogen infection in the small intestinal epithelium of the mouse. Significant and credible results in comparison to the control population (n=4 animals) are depicted as colored bars (Red: scCODA, purple: Dirichlet regression), stars depict the significance of the Poisson regression model carried out by Haber et al.[6] (*: adjusted $p<10^{-5}$, **: adjusted $p<10^{-10}$). The reference cell type for scCODA was determined automatically to be Endocrine cells (*Salmonella* (n=2 animals) and *H. polygyrus (Day10)* (n=2 animals)), and early transit-amplifying (TA Early) cells (*H.Polygyrus (Day 3)* (n=2 animals)), respectively. In all box plots, the central line denotes the median, boxes represent the interquartile range (IQR), and whiskers show values within. P-values and effect sizes are shown in **Supplementary Data 5.** In all box plots, the central line denotes the median, boxes represent the interquartile range (IQR), and whiskers show the distribution except for outliers. Outliers are all points outside 1.5 times of the IQR.

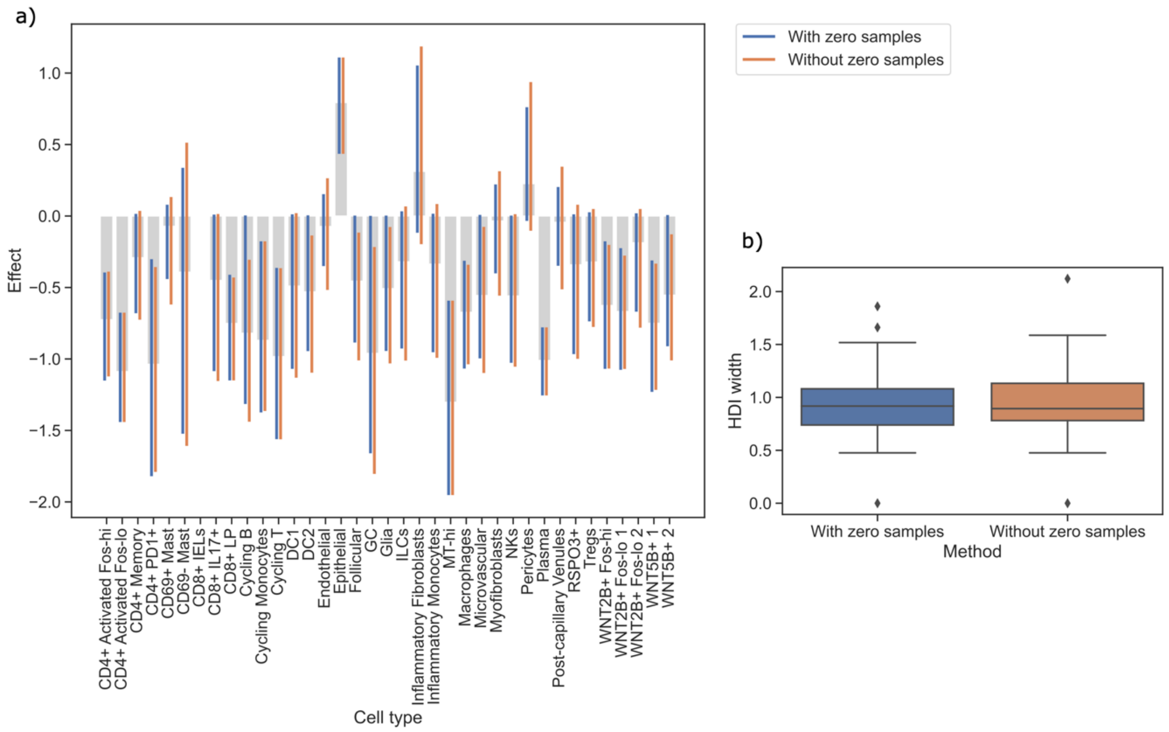

Supplementary Figure 11: High (95%) posterior density intervals (HDI) for effect parameters on ulcerative colitis data from the Lamina propria (Healthy (n=24 samples) vs. non-inflamed (n=24 samples)). We compare two ways of calculating the HDI - with (blue) and without (orange) including the MCMC samples where the corresponding spike-and-slab variable is zero. **(a)** Interval range (blue and orange bars) of both methods for each cell type. The grey bars show the posterior mean (including zero samples) for each effect. CD8+ IELs have no effect, since they were used as the reference. **(b)** Boxplots of HDI width (difference between upper and lower interval boundary) across all cell types. In the box plots, the central line denotes the median, boxes represent the interquartile range (IQR), and whiskers show the distribution except for outliers. Outliers are all points outside 1.5 times of the IQR.

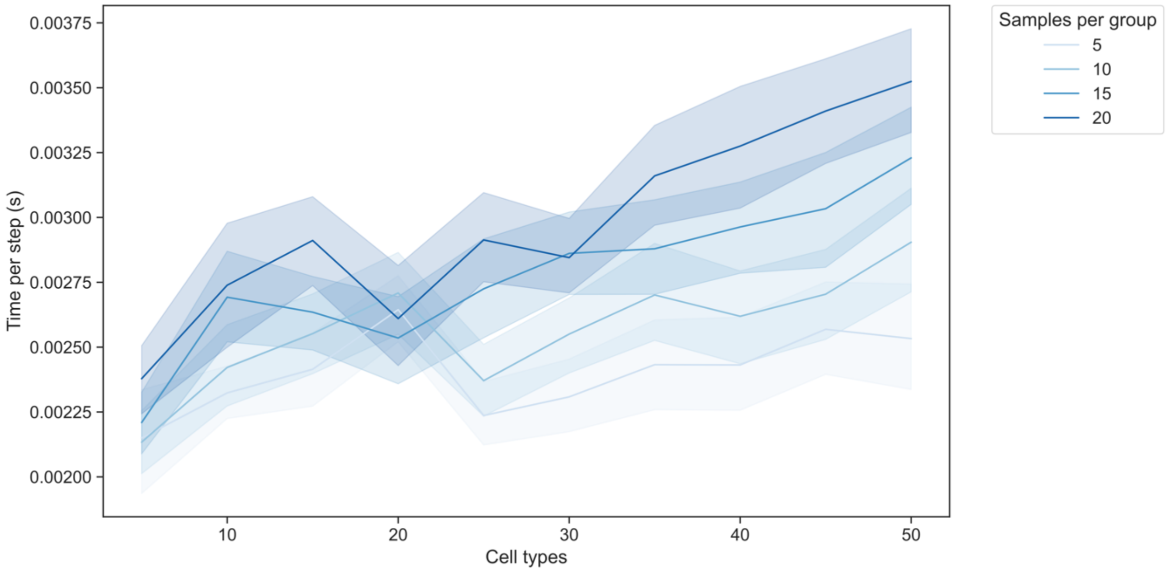

Supplementary Figure 12: Runtime analysis benchmark **(Methods - Runtime analysis)**. The time per HMC step (in seconds) is dependent on the number of cell types and the number of samples in the two treatment groups. The shaded areas depict the 95% confidence intervals around the mean. Generally, each HMC iteration takes longer for larger datasets. This effect is approximately linear in the number of cell types, with a less steep increase in runtime for datasets with fewer samples.

Supplementary Tables

Supplementary Table 1: Sensitivity analysis of differential abundance testing methods. AUC score from Receiver operating characteristic (**Fig. 2a**), Average precision score from precision-recall curve (**Fig. 2b**). All analyses were performed according to **Supplementary Table 2**.

**Method**	**ROC thresholding parameter**	**AUC**	**Average precision score**
scCODA	Inclusion probability	0.99	0.94
scDC	p-value	0.56	0.2
ANCOM	W-statistic	0.77	0.65
ALDEx2	p-value	0.9	0.77
ANCOM-BC	p-value	0.94	0.70
ALR+t-test	p-value	0.95	0.85
ALR+Wilcoxon	p-value	0.93	0.72
Dirichlet regression	p-value	0.7	0.31
Poisson regression	p-value	0.44	0.16
t-test	p-value	0.84	0.44
Beta-Binomial	p-value	0.84	0.41

Supplementary Table 2: Methods and configurations used in the benchmark comparison. Wrappers around implementations of all methods for easy use are implemented in the scCODA package. The Package column denotes the implementation that is called in scCODA.

**Method**	**Implementation details**	**Parameters**	**Package**
scCODA	Our proposed method	Reference cell type always set to the last component; FDR level 5%	scCODA package, version 0.1.3
Standard Dirichlet-Multinomial	Fully Bayesian model: Log-linear model on components of a Dirichlet-Multinomial distribution. Selection of a reference cell type. HMC inference setup identical to scCODA. Effects are credible if 0 is not included in the high-density interval	Reference cell type always set to the last component; High density interval: 95%	scCODA package, version 0.1.3
scDC	Single-cell differential composition analysis[7] Number of bootstrap samples generated for each data set: 100; no subject effects in linear model<br>*Note: This method did not give results for all datasets. The erroneous results were left out of the analysis*	False discovery rate: 5%	R-package scdney[7], version 0.1.5
ANCOM	Analysis of composition of microbiomes[14];<br>Used test: t-test; Holm-Bonferroni multiplicity correction (all recommended settings)	False discovery rate: 5%	Python-package scikit-bio[42], version 0.5.6

ALDEx2	ANOVA-Like Differential Expression tool for high throughput sequencing data[43]. Reference cell type set to the last component instead of the geometric mean; testing via t-test (Benjamini-Hochberg-corrected)	False discovery rate: 5%	R-package ALDEx2[15], version 1.22
ANCOM-BC	Analysis of compositions of microbiomes with Bias correction[13]; Holm correction of p-values (recommended)	False discovery rate: 5%	R-package ANCOMBC[13], version 1.0.5
ALR+t-test	Additive log-ratio transform of data; t-test (two-sided) on all components; Benjamini-Hochberg correction of p-values	Reference component: Last cell type; False discovery rate: 5%	Python-package scipy[44], version 1.6.1
ALR+Wilcoxon	Additive log-ratio transform of data; Wilcoxon-rank-sum test (two-sided) on all components; Benjamini-Hochberg correction of p-values	Reference component: Last cell type; False discovery rate: 5%	Python-package scipy[44], version 1.6.1
Dirichlet regression	Default settings: One-sample t-test of Dirichlet regression coefficients	Significance level 5%	R-Package DirichletReg [11], version 0.7
Poisson regression	Poisson regression model used by Haber et al. [6]; Benjamini-Hochberg correction of p-values	False discovery rate: (as used by Haber et al. [6])	Python-package statsmodels[45], version 0.12.1
t-test	t-test (two-sided) on all components of untransformed data; Benjamini-Hochberg correction of p-values	False discovery rate: 5%	scipy[44], version 1.6.1
Beta-Binomial	Variance estimation only for more than 2 samples per group possible; Test statistic: Likelihood-ratio (recommended for small sample sizes); Benjamini-Hochberg correction of p-values	False discovery rate: 5%	R-package corncob[16], version 0.2.0
