# Supplementary material for: scCODA is a Bayesian model for compositional single-cell data analysis
Source: Nat Commun. 2021 Nov 25;12:6876. doi: 10.1038/s41467-021-27150-6 (PMC8616929; doi:10.1038/s41467-021-27150-6)
Supplement: Supplementary file 2 — Description of Additional Supplementary Files [file 41467_2021_27150_MOESM2_ESM.pdf]

Description of Additional Supplementary Files

File Name: Supplementary Data 1
Description: Tabular results of the statistical analysis (scCODA, ANCOM, two-sided Wilcoxon rank-sum test with Benjamini-Hochberg correction of p-values) of the single-cell supercentenarian dataset by Hashimoto et al.

File Name: Supplementary Data 2
Description: Tabular results of the statistical analysis (scCODA, ANCOM) of the microglia dataset by Keren-Shaul et al.

File Name: Supplementary Data 3
Description: Tabular results of the statistical analysis (scCODA, two-sided t-test of Dirichlet regression coefficients with Benjamini-Hochberg adjusted p-values) of the lamina propria and epithelial cell composition by Smillie et al.

File Name: Supplementary Data 4
Description: Tabular results of the statistical analysis (scCODA, two-sample t-test, ANCOM) of the bronchoalveolar immune cell composition in COVID-19 patients by Liao et al.

File Name: Supplementary Data 5
Description: Tabular results of the statistical analysis (scCODA, ANCOM) of Haber et al. on the response to pathogen infection in the small intestinal epithelium of the mouse.
