# Supplementary material for: scCODA is a Bayesian model for compositional single-cell data analysis
Source: Nat Commun. 2021 Nov 25;12:6876. doi: 10.1038/s41467-021-27150-6 (PMC8616929; doi:10.1038/s41467-021-27150-6)
Supplement: Supplementary file 8 — Reporting Summary [file 41467_2021_27150_MOESM8_ESM.pdf]

nature portfolio

Corresponding author(s): Christian L. Müller and Benjamin Schubert

Last updated by author(s): Oct 25, 2021

Reporting Summary

Nature Portfolio wishes to improve the reproducibility of the work that we publish. This form provides structure for consistency and transparency in reporting. For further information on Nature Portfolio policies, see our Editorial Policies and the Editorial Policy Checklist.

Statistics

For all statistical analyses, confirm that the following items are present in the figure legend, table legend, main text, or Methods section.

n/a	Confirmed	
☐	☒	The exact sample size (*n*) for each experimental group/condition, given as a discrete number and unit of measurement
☐	☒	A statement on whether measurements were taken from distinct samples or whether the same sample was measured repeatedly
☐	☒	The statistical test(s) used AND whether they are one- or two-sided<br>*Only common tests should be described solely by name; describe more complex techniques in the Methods section.*
☐	☒	A description of all covariates tested
☐	☒	A description of any assumptions or corrections, such as tests of normality and adjustment for multiple comparisons
☐	☒	A full description of the statistical parameters including central tendency (e.g. means) or other basic estimates (e.g. regression coefficient) AND variation (e.g. standard deviation) or associated estimates of uncertainty (e.g. confidence intervals)
☐	☒	For null hypothesis testing, the test statistic (e.g. *F*, *t*, *r*) with confidence intervals, effect sizes, degrees of freedom and *P* value noted<br>*Give P values as exact values whenever suitable.*
☐	☒	For Bayesian analysis, information on the choice of priors and Markov chain Monte Carlo settings
☒	☐	For hierarchical and complex designs, identification of the appropriate level for tests and full reporting of outcomes
☒	☐	Estimates of effect sizes (e.g. Cohen's *d*, Pearson's *r*), indicating how they were calculated

Our web collection on statistics for biologists contains articles on many of the points above.

Software and code

Policy information about availability of computer code

Data collection	For the methods comparison and benchmark study, synthetic data was simulated using Python 3.8 and numpy = 1.19 and can be downloaded at https://doi.org/10.5281/zenodo.4305907. Publicly available datasets were downloaded form their respective repositories (see Data statement).
Data analysis	For analysis we used a custom Python library, scCODA available on Github at https://github.com/theislab/sccoda. The preprocessing of the single-cell datasets was performed using Scanpy. All scripts to reproduce the analyses can be found at https://github.com/theislab/scCODA_reproducibility.<br>The method has been implemented in Python 3.8 using Tensorflow = 2.3.2, Tensorflow-Probability = 0.11, ArviZ = 0.10, numpy = 1.19, Scanpy = 1.5, scikit-bio = 0.5.6, statsmodels = 0.12.1, scikit-learn = 0.24, scipy = 1.6.1 and R 4.1 using scdney = 0.1.5, ALDEx2 = 1.22, DirichletReg = 0.7, ANCOMBC = 1.0.5, corncob = 0.2.0. The Power Analysis was performed using caret package41 (R 4.1).

For manuscripts utilizing custom algorithms or software that are central to the research but not yet described in published literature, software must be made available to editors and reviewers. We strongly encourage code deposition in a community repository (e.g. GitHub). See the Nature Portfolio guidelines for submitting code & software for further information.

Data

Policy information about availability of data

All manuscripts must include a data availability statement. This statement should provide the following information, where applicable:

- Accession codes, unique identifiers, or web links for publicly available datasets
- A description of any restrictions on data availability
- For clinical datasets or third party data, please ensure that the statement adheres to our policy

The synthetic benchmark datasets and results are available at https://doi.org/10.5281/zenodo.4305907. The single-cell datasets can be found in their respective public repositories. The supercentenarians PBMC dataset by Hashimoto et al. can be found at http://gerg.gsc.riken.jp/SC2018, while the Alzheimer's mouse microglia dataset by Keren-Shaul et al. can be accessed at GEO under GSE98969 (https://www.ncbi.nlm.nih.gov/geo/query/acc.cgi?acc=GSE98969). The single-cell ulcerative colitis dataset by Smilie et al. can be downloaded from the Single-Cell Portal (Accession ID SCP259; https://singlecell.broadinstitute.org/single_cell/study/SCP259/intra-and-inter-cellular-rewiring-of-the-human-colon-during-ulcerative-colitis) and its accompanying analysis code and description from https://github.com/cssmillie/ulcerative_colitis. The processed single-cell data of bronchoalveolar immune cells in patients with COVID-19 by Liao et al. is publicly available at https://github.com/zhangzlab/covid_balf. The single-cell data of small intestinal epithelium cells infected with different bacteria is available from Single Cell Portal (accession ID SCP44, https://singlecell.broadinstitute.org/single_cell/study/SCP44)

Field-specific reporting

Please select the one below that is the best fit for your research. If you are not sure, read the appropriate sections before making your selection.

☒ Life sciences ☐ Behavioural & social sciences ☐ Ecological, evolutionary & environmental sciences

For a reference copy of the document with all sections, see nature.com/documents/nr-reporting-summary-flat.pdf

Life sciences study design

All studies must disclose on these points even when the disclosure is negative.

Sample size	For simulated data, we chose sample size according to common single-cell RNAseq experiments (2-20) to estimate the effect of sample size on identifying the correct effects. For actual single-cell RNAseq data, we used published datasets. To this end, we collected a non-exhaustive list of single cell experiments which performed compositional analysis and reanalyzed the experiments using the complete datasets.
Data exclusions	From published datasets, we used the full dataset and selected only datasets with several replicates.
Replication	Simulation experiments were re-run multiple times to verify the robustness of the statistical inference. Source code (https://github.com/theislab/scCODA_reproducibility) and datasets (https://doi.org/10.5281/zenodo.4305907) of all experiments are provided for reproducibility. From published datasets, we chose only datasets with several replicates.
Randomization	Does not apply for the manuscript as we worked with published data and did not conduct any studies ourselves.
Blinding	Does not apply to used real-world data, as we worked with already published datasets and did not conduct any studies ourselves.<br><br>To benchmark scCODA and other statistical tests used for compositional data analysis, we generated synthetic datasets following realistic noise distributions of single-cell experiments with increasing effect sizes and sample sizes to estimate the performance of the different statistical tests. No information was given to the different models which of the simulated cell types were artificially shifted.

Reporting for specific materials, systems and methods

We require information from authors about some types of materials, experimental systems and methods used in many studies. Here, indicate whether each material, system or method listed is relevant to your study. If you are not sure if a list item applies to your research, read the appropriate section before selecting a response.

Materials & experimental systems

n/a	Involved in the study
☒	☐ Antibodies
☒	☐ Eukaryotic cell lines
☒	☐ Palaeontology and archaeology
☒	☐ Animals and other organisms
☒	☐ Human research participants
☒	☐ Clinical data
☒	☐ Dual use research of concern

Methods

n/a	Involved in the study
☒	☐ ChIP-seq
☒	☐ Flow cytometry
☒	☐ MRI-based neuroimaging
